# Antibacterial isoamphipathic oligomers highlight the importance of multimeric lipid aggregation for antibacterial potency

**DOI:** 10.1038/s42003-018-0230-4

**Published:** 2018-12-07

**Authors:** Joseph S. Brown, Zeinab J. Mohamed, Christine M. Artim, Dana N. Thornlow, Joseph F. Hassler, Vincent P. Rigoglioso, Susan Daniel, Christopher A. Alabi

**Affiliations:** Robert Frederick Smith School of Chemical and Biomolecular Engineering, 120 Olin Hall, Cornell University, Ithaca, NY 14853 USA

**Keywords:** Biophysical chemistry, Membranes, Antimicrobials, Biophysical chemistry

## Abstract

Cationic charge and hydrophobicity have long been understood to drive the potency and selectivity of antimicrobial peptides (AMPs). However, these properties alone struggle to guide broad success in vivo, where AMPs must differentiate bacterial and mammalian cells, while avoiding complex barriers. New parameters describing the biophysical processes of membrane disruption could provide new opportunities for antimicrobial optimization. In this work, we utilize oligothioetheramides (oligoTEAs) to explore the membrane-targeting mechanism of oligomers, which have the same cationic charge and hydrophobicity, yet show a unique ~ 10-fold difference in antibacterial potency. Solution-phase characterization reveals little difference in structure and dynamics. However, fluorescence microscopy of oligomer-treated *Staphylococcus aureus* mimetic membranes shows multimeric lipid aggregation that correlates with biological activity and helps establish a framework for the kinetic mechanism of action. Surface plasmon resonance supports the kinetic framework and supports lipid aggregation as a driver of antimicrobial function.

## Abstract (web summary)

Joseph Brown et al. use oligothioetheramides (oligo TEAs) to show that multimeric lipid aggregation in *Staphylococcus aureus* mimetic membranes correlates with the biological activity of oligoTEAs. These results may explain why antimicrobial peptides with identical cationic charge and hydrophobicity show different biological activity.

## Introduction

Antibiotic resistance continues to grow as a world health crisis due to the natural evolution of bacteria, an increase in antibiotic use and accessibility, as well as a substantial decline in development^1,2^. As resistance accumulates, researchers have turned to the development of new antibiotic strategies including cationic antimicrobial peptides (AMPs)^3^. As a structurally broad class, AMPs encompass short, amphipathic peptide units that share common cationic and hydrophobic features. They naturally serve as a part of the innate immune system and are an evolutionarily conserved response to foreign pathogens^4,5^. Several modes of action have been proposed, with some AMPs acting by disrupting and permeabilizing the bacterial membrane^6,7^. Additional steps can contribute to bacterial death including membrane polarization, disruption of cytoplasmic components, immunomodulatory response, or adjuvant function^8–10^. As the bacterial membrane is essential and AMP disruption is diverse and stochastic, bacteria can have difficulty circumventing this mechanism^6,11^. Understanding the fundamental mechanism of antibiotic action is critical, considering that almost every new antibiotic produced in the past 60 years has succumbed to bacterial resistance within a few years of release^12^.

AMPs have encountered barriers to their systemic use, predominantly due to their toxicity, proteolytic degradation, and low bioavailability^4,8,13^. Several AMPs and lipophilic AMPs including polymixin B, nisin, gramicidin S, and colistin have been developed, but relegated to topical application, food packaging, or as drugs of last resort, with the exception of daptomycin^4,14–17^. Toxicity is primarily due to insufficient selectivity, where AMPs generally interact more strongly with bacterial membranes based on their lipid composition and properties. Anionic lipids within bacterial membrane are broadly targeted by AMPs over the more neutral and rigid cholesterol-containing mammalian membrane^5,18^. Low bioavailability is primarily due to rapid proteolytic degradation of peptides by serum proteases. Using this knowledge, researchers have worked toward enhancing serum stability with the development of sequence-defined peptidomimetics including peptoids^19^, β-peptides^20–22^, oligothioetheramides (oligoTEAs)^23,24^, and many others. To control activity and selectivity, AMPs and AMP mimetics have focused on tuning the nature, quantity, and spatial positioning of cationic charge and hydrophobicity^4,6,10,13^. This optimization generally holds, even within structured macromolecules containing α-helices, where these fundamental properties lead to interfacial amphipathicity^25–27^. Thus, with these design parameters, some AMPs and AMP mimetics with promising prospects have been developed (e.g., brilacidin in Phase II clinical trial)^28,29^.

Researchers have also established limitations within the optimization of potency and selectivity using cationic charge, hydrophobicity, and amphipathicity^6,10^. For example, hydrophobicity improves membrane insertion and potency, but can increase both in vitro and in vivo toxicity^19,23,30–32^. Similar trends have been seen for the level of amphipathicity^25^. Thus, researchers have called for the advancement of design principles to include targeting strategies or biophysical parameters^6,33,34^. Beyond these common molecular-scale physicochemical properties, biophysical characterization potentially holds the next level of parameters to direct the design of therapeutically relevant membrane-disrupting antimicrobials^6,7^. Several biophysical techniques probe the interaction of these membrane disruptors with supported bacterial mimetic bilayers including surface plasmon resonance (SPR)^35,36^, quartz crystal microbalance^37,38^, and more recently dual polarization interferometry^7,39^. Thus far, these studies have revealed a complex sequence of events that are often indistinguishable including binding, insertion, and structural changes made to bacterial membranes by disruptive AMPs and their mimetics^35,36^. Moreover, these studies have developed the concept of a critical threshold concentration of membrane disruption, argued to direct the minimum inhibitory concentration (MIC)^6^, or another concentration in which irreversible structural changes are made to the membrane^7^.

Toward understanding new parameters for AMP optimization and development, we have explored a unique pair of sequence-defined oligoTEA constitutional isomers of the same length, cationic charge, hydrophobicity, and thus amphipathicity^23^. These antibacterial oligoTEAs (AOTs) have the same physical and chemical properties that typically guide optimization of membrane-disrupting antimicrobials, but have displayed a unique differential in potency and toxicity of nearly tenfold. Thus, they were ideal for exploring new parameters for sequence–structure–function optimization of membrane-disrupting antimicrobials. We confirmed similar solution-phase structures using small- and wide-angle X-ray scattering (SAXS/WAXS), as well as pulsed field gradient (PFG) nuclear magnetic resonance (NMR) and pulsed electron paramagnetic resonance (EPR) processed within the molecular Stokes–Einstein–Sutherland (SES) relation^40^. However, directed by differences in biophysical observations, we explored the interaction of these oligomers with supported bacterial mimetic bilayers using fluorescence microscopy, fluorescence recovery after photobleaching (FRAP), and oligomer–lipid extraction. All biophysical experiments created a de novo kinetic framework that was then tested and supported by modeling oligomer–membrane interactions observed by SPR. Thus, we are able to present new parameters that can enable further development of membrane-disrupting antibacterial agents beyond typical physicochemical parameters of cationic charge and hydrophobicity.

## Results

### Different potency yet similar solution-phase structures

**Fig. 1:**
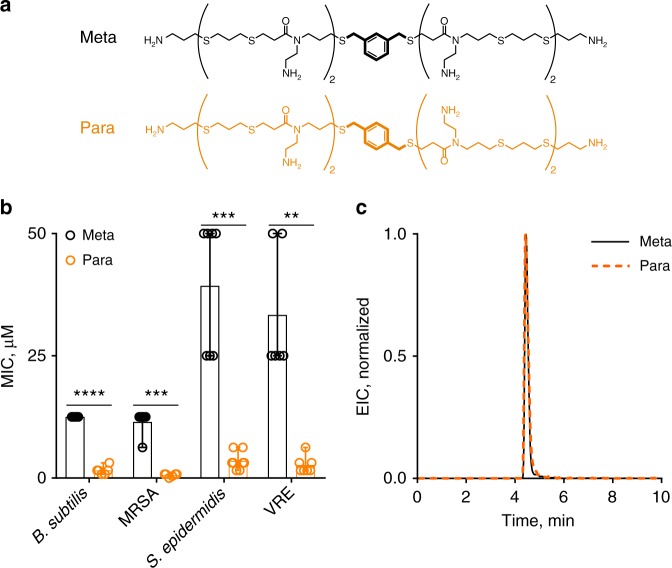
Antibacterial and hydrophobicity characterization. **a** Structures of the Meta and Para. **b** Minimum inhibitory concentration (MIC) results showed nearly a tenfold difference in potency across several clinically relevant bacterial strains. Error bars represent the range of the MIC observed. (*n* = 3 biological replicates each with *n* = 2 technical replicates shown). See Supporting Information for MIC definition (Supplementary Figure 1). MRSA is methicillin-resistant *Staphylococcus aureus* and VRE is vancomycin-resistant *Enterococcus*. Significant difference in potency: *p* < 0.0001, 0.0002, 0.0005, and 0.0021 paired *t*-test, two-tailed, *df* = 5 for all, *B. subtilis*, MRSA, *S. epidermidis*, and VRE, respectively. **c** Reverse-phase HPLC-MS chromatogram of the extracted product mass (EIC) of the Meta and Para demonstrated the same retention and hydrophobicity

Membrane-disrupting antimicrobials make use of cationic charge and hydrophobic moieties to bind and insert into bacterial membranes, respectively^4,6,13^. In previous work, we examined structural features such as oligomer length (total charge), hydrophobicity, sequence, and composition^23,24,32^. Relationships observed between chemical and physical properties with activity corroborate conclusions made across multiple molecular classes: a threshold of cationic charge is required for activity and hydrophobicity increases potency while increasing toxicity. However, exceptions were found based on the conformation of a benzyl group at the center of the first-generation AOT scaffold (Fig. 1a). The oligomer with a para-substituted benzyl group (“Para”) showed nearly an order of magnitude higher potency than the meta-substituted version (“Meta”) against several clinically relevant pathogens as measured by a MIC assay (Fig. 1b, see Supplementary Figure 1 for MIC definition). The MIC of the Meta and the Para were recorded as the minimum concentrations that prevented 90% of visible bacterial growth, also called a MIC_90_. A similar trend was observed with toxicity as measured by the hemolysis assay (Supplementary Figure 2). However, these oligomers showed the exact same hydrophobicity as demonstrated by their retention on reverse-phase high-performance liquid chromatography (HPLC) (Fig. 1c) and are thus isoamphipathic.

**Fig. 2:**
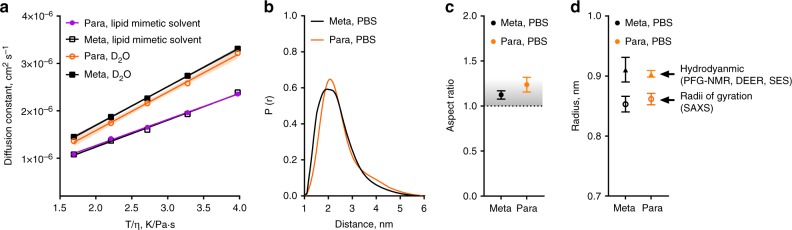
The Meta and Para have highly similar solution-phase structures. **a** Variable temperature PFG-NMR (1 mM) completed from 0 to 40 °C in D_2_O and a lipid mimetic solvent (1:4:4 D_2_O:dMeOD:CDCl_3_) shown and shaded regions indicating SD. The diffusion constant is plotted vs. the viscosity (*η*) normalized temperature. Statistical significance for D_2_O: identical slopes, *p* = 0.862, two-tailed *t*-test and for lipid mimetic solvent: identical slopes, *p* = 0.485, two-tailed *t*-test. **b** Reconstructed end-to-end distance profiles from DEER EPR of samples rapidly vitrified to 70 K from room temperature (RT, 22 °C) 50 μM in PBS. **c** Aspect ratio calculated from diffusion and end-to-end distance measurements and a molecular Stokes–Einstein–Sutherland (SES) equation at RT. The shaded region reflects the aspect ratio of an ideal, well-solvated polymer (AR ~ 1.1). **d** The calculated hydrodynamic radii from the SES equation and the radii of gyration for the Meta and Para from Guinier fits of SAXS data (see Supplementary Figure 5 and S6 for raw data). In **c** and **d**, error bars represent SD, propagated from the PFG NMR measurement and the DEER end-to-end distance

As these isomeric oligoTEAs have different connectivity, their solution-phase structure was initially suspected to be responsible for the differences seen in their antimicrobial potency. Solution-phase size, shape, and conformation were examined by PFG NMR, double electron–electron resonance (DEER) EPR, and SAXS (Fig. 2). PFG NMR is a label-free technique to measure the self-diffusion of molecules observed by NMR within the solution-phase, encoding information about the hydrodynamic size and shape. Variable temperature (VT) PFG NMR revealed similar diffusion coefficients across a range of viscosity-normalized temperatures (identical slopes, *p* = 0.862, two-tailed *t*-test) for both isomers. This linearity indicated minimal intermolecular interactions or intramolecular transitions during measurement, which would appear as functions of temperature (e.g., aggregation, repulsion). To gauge the solution-phase structure at the lipid membrane, VT PFG NMR was also performed in a lipid mimetic solvent of 1:4:4 water:methanol:chloroform, preivously used for structure determination of transmembrane proteins^41,42^. In this lipid mimetic solvent, both the Meta and Para present similar diffusion characteristics (identical slopes, *p* = 0.485, two-tailed *t*-test). The oligomers show slower diffusion, likely due to expanded hydrodynamic radii, a sign of improved solvation. This expansion and solvation could indicate an entropic gain if the oligomer were to bind and then insert into the hydrophobic space in the lipid bilayer.

To investigate the dynamics of these oligoTEAs, DEER EPR was performed. End-to-end distance measurements by DEER measures the dipolar coupling between two unpaired electrons from “spin” probes on the molecule using pulsed EPR. Reconstruction of the observed data extracts a distance distribution between the spin probes. The di-spin labeling of the oligomers was made possible by use of a phthalimide protected *N*-allyl-*N*-acrylamide monomer during oligomer assembly. Thus, only the terminal amines were di-spin labeled with a proxyl nitroxide (Supplementary Figure 3–4). Deprotection of the di-spin-labeled oligoTEA *N*-phthalimides was optimized using a sodium borohydride reduction and acid hydrolysis to provide the final di-spin-labeled Meta and Para (Supplementary Figure 35–36)^43^. DEER measurement was completed on 50 µM oligomer after rapid vitrification from room temperature (RT) in phosphate-buffered saline (PBS) with 10 v/v% ethylene glycol. Reconstructed distance distributions revealed similarities in both end-to-end distance distributions (Fig. 2b).

Together, the end-to-end distance from DEER and diffusion measurements enable calculation of the hydrodynamic radius and aspect ratio with the SES equation (Fig. 2c, d; Supplemental Methods). This calculation uses the slope of the plot of diffusion vs. viscosity-normalized temperature (Fig. 2a), and allows structural elucidation of highly flexible, small structures^40^. The calculated hydrodynamic radii were under 1 nm and the aspect ratios reveal similar spherical oligoTEA shapes (Fig. 2c, d). Spherical appearance of the oligomer indicated clear space averaging during observation from single-chain collapse. These dynamics were also visualized in a similar broad DEER distribution (full-width at half-maximum of ~ 2 nm). These data from DEER demonstrate that the oligomers have similar dynamics due to the same size and distribution, likely indicating similar flexibilities.

To complement the SES analysis, X-ray scattering was completed to confirm similarities in the Meta and Para oligomers and verify the SES-calculated hydrodynamic radii (Supplementary Figure 5). When done in the solution phase, SAXS and WAXS is a label-free technique that encodes information about macromolecular size, shape, and dynamics by measuring the scattering of X-rays from a sample. Scattering was examined at both small- and wide angle due to the small size of these oligomers to measure their radii of gyration^44,45^. Broad scattering was observed decaying toward the baseline around *q* ~ 0.4–0.5 (Supplementary Figure 5a). Guinier fits provided the radii of gyration in good agreement with the SES analysis at ~ 0.5 Å below the hydrodynamic radii, expected from oligomer hydration (Supplementary Figure 5b, Supplementary Table 1). Moreover, exceptional overlap in calculated SAXS pair-wise distributions was observed between the Meta and Para (Supplementary Figure 6). Overall, these results demonstrate remarkable similarities between the highly dynamic and spherical solution-phase structures of the Para and Meta oligoTEAs, strongly suggesting they are indistinguishable in solution. Thus, these flexible and dynamic oligomers likely initiate their interaction with the bacterial membrane in highly similar manners. Traditionally, membrane-disrupting AMPs and mimetics initiate this binding via electrostatic interactions, but their composition and structure influences the thermodynamics and kinetics of the interaction. Structure in solution is not a prerequisite for activity, however, and structure can develop within subsequent binding states^14,35,46^.

### Meta and Para were distinguishable at the bacterial membrane surface

**Fig. 3:**
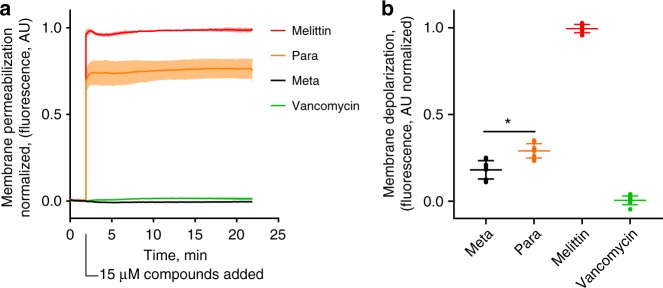
The Meta and Para were distinguishable and active on the bacterial membrane. **a** Propidium iodide (PI) assay demonstrated the ability of the Para to disrupt and permeabilize the bacterial membrane of MRSA (ATCC 33591) at significantly greater activity than the Meta (*p* = 0.0015, df = 2.018, unpaired *t*-test, two-tailed). The shaded region indicates the standard deviation around a solid line (mean) of *n* = 3 biological replicates with *n* = 2 technical replicates each. **b** diSC_3_5 membrane depolarization assay performed against MRSA *(n* = 3 biological replicates). The Para shows stronger membrane depolarization, (*p < 0.028, df = 3.995, unapaired *t*-test, two-tailed). In both assays, oligomers and controls were added at 15 μM to compare their activity. Vancomycin inhibits cell wall synthesis and serves as a negative control

As the mechanism of AMPs and AMP mimetics includes membrane disruption^7,23^, additional biological testing was pursued on the mechanism of the Meta and Para. A propidium iodide (PI) assay was completed on methicillin-resistant *Staphylococcus aureus* MRSA. PI is a membrane-impermeable agent that fluoresces upon binding and intercalation with nucleic acid. Thus, PI displays fluorescence if the bacterial membrane is permeabilized to allow either diffusion of PI into the bacteria or diffusion of nucleic acid out of the bacteria. The PI assay demonstrate that the Para rapidly compromised the bacterial membrane (Fig. 3a), with significantly higher permeabilization shown by the Para than the Meta as expected. The difference between the Meta and Para is similar when tested with *Bacillus subtilis* (Supplementary Figure 7). Membrane depolarization was measured with fluorescent 3,3′-dipropylthiadiacarbocyanine (diSC_3_5), which binds and quenches at the membrane surface from the natural bacterial polarization. Membrane disruption leads to depolarization, releasing, and dequenching diSC_3_5^47^. Using this assay, the Para shows a greater extent of depolarization than Meta (Fig. 3b), with less of a difference than the PI assay. Both the PI and diSC_3_5 assays demonstrated results expected for membrane-disrupting agents such as melittin. These results also corroborated the differences between the Meta and Para, despite their extensive similarities in solution-phase structure, size, charge, and hydrophobicity.

### Meta and Para triggered lipid surface aggregates formation

As the Meta and Para presented distinguishable membrane interactions, we sought to investigate their interaction and disruption on a *S. aureus* mimetic supported lipid bilayer (SLB) via fluorescence microscopy. Bacteria innately have different lipid compositions within their membranes, which can influence the interaction and effectiveness of AMPs. As MRSA results in a significant percentage of deaths from antibiotic-resistant bacteria^48^, we focused on simulating its lipid membrane composition. A head group composition of 4:5:11 neutral lipids, cardiolipin, and phosphatidylglycerol was compiled from literature on the *S. aureus* membrane (see Supporting Information). Lipids tail groups were chosen to maintain fluidity at RT, specifically neutral 18:1 diacylglycerol, tetrapalmitoyl cardiolipin, and 1-palmitoyl-2-oleoyl-sn-glycero-phosphoglycerol. Small unilamellar vesicles (SUVs) were prepared with this composition and labeled with Octadecyl Rhodamine (R18). SLB preparation was enabled by pre-coating glass slides with poly-l-lysine (PLL)^49^ (see Supplementary Methods, Supplementary Figure 8). The resulting lipid bilayers were visualized and FRAP was completed to verify their quality and fluidity (Supplementary Figure 9–10). FRAP can determine the lateral diffusion of two-dimensional lipid bilayers, even on living cellular membranes, by observing the recovery of a photobleached spot.

**Fig. 4:**
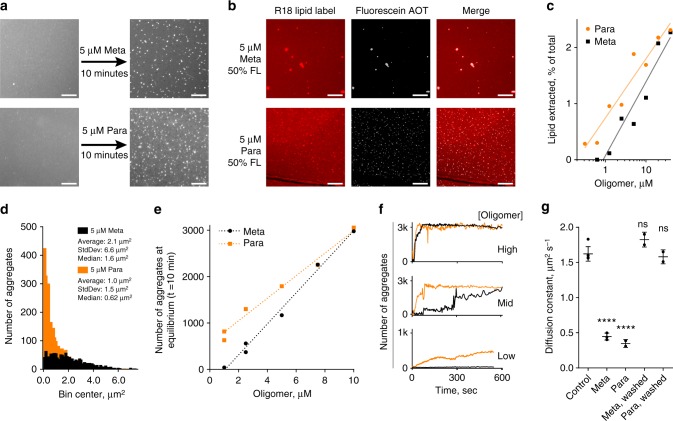
Visualization of aggregate count, size, and kinetic formation, as well as evidence of membrane loss and binding. **a** Fluorescent micrographs of before and after 10 min of oligomer exposure, R18 visualizes the lipids. **b** Use of fluorescein Meta and Para demonstrated oligomer participation via colocalization in the aggregate (10 min, 5 μM total, 50 %FL, washed with PBS). All scale bars are 25 μm. **c** Capture of lipid particles evolved from surface quantified by plate reader fluorescence as a percentage of total within the microscopy well. Semi-log lines are an aid to the eye (*n* = 1). **d** Histogram of aggregate sizes on microscopy images determined by ImageJ particle analysis. **e** Total number of aggregates in microscopy image after 10 min vs. oligomer concentration showed a threshold of efficiency. A linear regression is shown to aid the eye. At high (active) oligomer concentration the oligomers were indistinguishable; at low concentration the Para developed more aggregates, as it is more potent. Individual SLBs were observed (*n* = 1) for all concentrations except 1 μM Meta and 2.5 μM Para, where *n* = 2. **f** Kinetics of aggregate formation showing the oligomers are indistinguishable at high concentration, but distinguishable at intermediate and low concentrations. 10 μM, 7.5 μM, and 1 μM are the high, mid, and low concentrations, respectively. Note for 1 μM the *y* axis is 1k, whereas for 10 μM and 7.5 μM the scale is 3k aggregates. **g** FRAP experiment revealing reversible decrease in membrane diffusivity when equilibrated with 10 μM oligomer from a molecular, non-aggregate state (****p < 0.0001; ns *p* > 0.05, unpaired *t*-test, two-tailed). FRAP replicates represent membrane heterogeneity, *n* = 4,3,2,2 for the Control, Meta, Para, Meta Washed, and Para Washed, respectively (see Supplementary Figure 18 for mobile fraction).

Treatment of the *S. aureus* mimetic membranes for 10 min with 5 µM Meta and Para revealed the formation of micrometer-sized lipid aggregates and lipid particle evolution from the surface into the bulk solution (Fig. 4a, Supplementary Movie 1 and 2, Supplementary Figure 10). Treatment at 5 µM was chosen as it is in between the MIC of the oligomers to discern any difference. Control experiments demonstrated the phenomena was not specific to the R18 label. SUVs prepared with Texas Red^TM^ DHPE in place of R18 revealed similar lipid aggregation and particle evolution phenomena (see Supplementary Movie 3).

To further understand their participation in membrane disruption, the Meta and Para were fluorescently labeled to visualize oligomer colocalization with the aggregates. Fluorescein acrylamide was conjugated to the oligoTEAs via the terminal thiol at the end of oligomer assembly. Following acid cleavage, the final product was purified by HPLC and verified via ^1^H NMR and liquid chromatography–mass spectrometry (LCMS) (Supplementary Figures 11, 37–40). The fluorescein-labeled Meta and Para were mixed with unlabeled oligomer and placed onto *S. aureus* mimetic membrane using the same conditions (5 μM, 10 min, RT, 50% labeled). Due to the fluorescence of the solution, imaging was completed after buffer exchange with PBS to remove solution-phase and loosely bound fluorescein-labeled oligomers. The fluorescent microscopy images showed that the fluorescein-labeled Meta and Para were colocalized with the lipid aggregate, likely directing its formation (Fig. 4b). In addition, the biological activity was also checked, revealing decreased potency and differential between the Meta and Para after fluorescein labeling (Supplementary Figure 12). Thus, the fluorescein oligomers did not fully represent the mechanism of action of the unlabeled oligomers, but all shared the physical phenomena of aggregate formation. Fewer aggregates were observed relative to the unlabeled oligomers with size differences, likely because of the attenuated biological activity.

Lipid removal from the SLB membrane was confirmed by measuring the fluorescence of bulk solution in the microscopy well via R18. The *S. aureus* mimetic SLBs labeled with R18 were incubated with the oligomers for 5 min, as time-lapse imaging showed the lipid particles could settle after 7–10 min. The bulk solution was removed and treated with Triton-X detergent at 5 mM final concentration to solubilize any lipid particles^50^. The remaining SLB membrane in the microscopy well was also extracted fully by treatment with Triton-X detergent (verified in Supplementary Figure 13). Comparison of the fluorescence revealed 1–2.5% of the membrane is extracted into the bulk solution during oligomer exposure over a concentration range of 0.25–40 μM (Fig. 4c). The Para showed more extraction at lower concentrations. Moreover, lipid extraction was observed below concentrations where lipid particles were observed in the fluorescence microscopy, indicating lipid extraction can occur in smaller, non-aggregate forms (e.g., micelles).

Analysis of the aggregates at equilibrium revealed the Para forms smaller and more numerous micrometer-sized aggregates at lower concentrations (Fig. 4d, e). A higher number of aggregates formed per oligomer concentration defines efficient aggregate formation. ImageJ particle analysis revealed the Para and Meta form aggregates at similar rates and efficiency at higher concentrations of ≥ 7.5 μM (Fig. 4e, f), closer to where both oligomers are antimicrobial (see Supplementary Methods, Supplementary Figure 14–15). However, at lower concentrations of ≤ 5 μM, the Para remains active against MRSA and retains efficient aggregate formation, whereas the Meta is inactive and shows attenuated aggregate production (Fig. 4e). Efficient aggregate formation appears to have a concentration threshold in similar style to the MIC and could be important for oligomer antimicrobial function. However, the MIC_Meta_ is much higher than this concentration, undermining any immediate quantitative correlation, likely because of differences in experimental set up. With regards to size, a smaller size aggregate increases surface density, possibly increasing any antimicrobial action of the aggregate per oligomer mass. The smaller size or the efficiency of aggregate formation could be a potential explanation as to why the Para is much more potent than the Meta. Overall, the particle count and size data obtained from fluorescence microscopy show differences between the Para and Meta that could correlate with their differences in antimicrobial activity.

### Kinetic mechanism proposed from microscopy observations

Time-lapse fluorescence microscopy of oligomer disruption of the *S. aureus* mimetic SLB clearly showed irreversible aggregate formation, where few to no aggregates re-adsorbed into the mobile lipid bilayer after their formation or washing. Analysis of aggregate count in all frames over time revealed kinetic behaviors that are dependent on oligomer concentration, supplementing evidence of a threshold in similar style to the MIC (Fig. 4f). At high concentration, both oligomers exhibited quick aggregate formation kinetics to a level of ~ 3k particles, or ~ 20k particles per mm^2^. At intermediate concentration, aggregate formation from the Para quickly reached an equilibrium aggregate density, whereas the Meta is slow. At low concentrations, the Para shows slower aggregate formation, whereas the Meta shows little to no aggregate formation. Overall, these data indicated concentration dependence on kinetic formation of the aggregate, also in a similar style to the biological activity (MIC).

FRAP of the *S. aureus* mimetic SLB membrane revealed evidence of sub-micrometer, reversible oligomer binding, in contrast to the irreversibly formed aggregate. Treatment of the membrane with the Meta and Para showed a decrease in diffusivity by FRAP (Fig. 4g). The aggregates only contributed ≤ 4% of the FRAP laser spot size (Supplementary Figure 16 and Supplementary Methods for calculation). Therefore, the decreased diffusion measured by FRAP represented changes in the membrane not due to the immobile aggregates. At 10 µM, there is not a large difference between the Meta and Para, also in contrast to the aggregate, indicating the sub-micron binding events progress to a similar equilibrium. The SLB was thoroughly washed with PBS to remove solution-phase and loosely bound oligomer. Afterward, FRAP shows the membrane diffusivity returned to its native state, indicating this binding was reversible (Fig. 4g). Additional oligomer concentrations of 2.5 and 5 µM were also tested (Supplementary Figures 17, 18). At these lower concentrations, the decrease in diffusion lessened in a concentration-dependent manner and binding remained reversible. Molecular binding states that reversibly changed the membrane diffusivity could be the oligomer crosslinking the phospholipid head-groups or inserting into the membrane to cause viscoelastic changes. As described in literature, there are several hypothesized molecular- and macromolecular-scale binding states seen with AMPs including “bind and insert” mechanisms, which would not be visible by microscopy^7,10,13,35^.

**Fig. 5:**
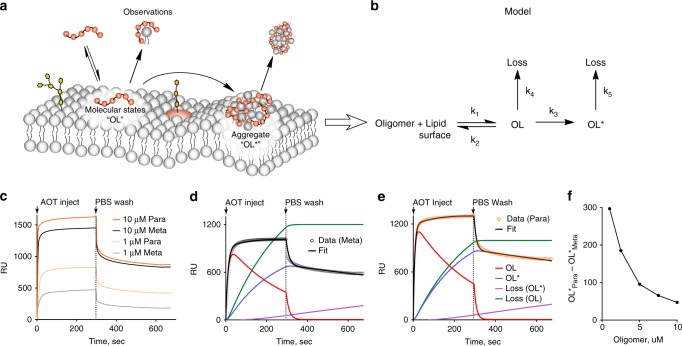
Kinetic model based on observations tested and supported by SPR. **a** Kinetic framework was developed from all observations from time-lapse fluorescence microscopy (aggregates, lipid particle evolution), lipid extraction experiments (nanoscale losses), and FRAP (molecular-scale binding) and translated into **b** a testable hypothesis of oligomer-membrane disruption. **c** SPR sensorgram of a high (10 μM) and low (1 μM) concentration. The Meta and Para showed similarities at high concentration, especially within the dissociation phase (PBS wash). At low concentrations, the oligomers were significantly different in both the association and dissociation phases. All runs did not return to the original baseline, with some irreversible changes. **d** Model fit of 5 μM Meta. **e** Model fit of 5 μM Para. **f** Concentration dependence of differential aggregate formation (OL*), where the Para showed higher activity at lower concentration, below the MIC of the Meta

All data from the fluorescence microscopy and FRAP guided the development of a kinetic framework around the mechanism of the Meta and Para membrane disruption. The FRAP showed molecular-scale states that bind reversibly. These reversible states reached equilibrium faster than the FRAP time-scale (minutes), but binding could be quick if initiated by electrostatic interactions. Aggregate formation is irreversible and entraps oligomer onto the membrane surface, occurring after molecular binding. Lipid losses from particle or micelle formation likely includes oligomer as well, as some aggregates are observed dissociating from the membrane surface via a meta-stable “worm” (see Supplementary Movies). Altogether, these observations begin to describe both the kinetics and mechanism of the oligomers’ membrane-disrupting action (Fig. 5a).

### Kinetic hypothesis supported by SPR

With a hypothesized kinetic framework (Fig. 5a), SPR tested the kinetic model against observed data of *S. aureus* mimetic membrane disruption by the oligomers (Fig. 5b). SPR is a label-free technique that uses an optical biosensor to observe effective refractive index changes near the gold sensor surface. It encodes both mass and structural changes, making it ideal to observe biomolecular interactions, such as membrane disruption, in real-time. Using a Biacore L1 chip^36,51,52^, *S. aureus* mimetic membranes were formed on the sensor and the Meta and Para were injected to observe the kinetics of binding and membrane disruption (Supplementary Figure 19). Upon injection of oligomer, all response curves ascended quickly and then slowed toward an equilibrium, consistent with two-phase binding kinetics (Fig. 5c). Specifically, it appears oligomer was quickly bound to the lipid membrane (OL) and then shifted into another second, subsequent bound state (OL*). After the membrane was washed with PBS, a quick drop to an elevated baseline was observed to indicating the presence of both reversible and irreversible states (Fig. 5c, Supplementary Figure 20).

The proposed model based on fluorescence microscopy, lipid extraction, and FRAP fit well to the experimental SPR data, unlike literature models (Fig. 5b, Supplementary Methods, Equations S1). Several models have been developed for AMPs binding to SPR mimetic membranes, commonly utilizing two reversible steps including loss or lipid expansion on the second step^35,51,52^. However, attempts to fit the SPR sensorgrams with literature-based two-state models with or without loss on the second step were unsuccessful (Supplementary Figure 21–22). The model could fit parts of the sensorgram (e.g., association, dissociation phase), but not the complete curve. Addition of loss on the first step (OL) to the model successfully described the SPR data (Fig. 5c). This loss was rationalized by the sub-micrometer losses observed in the lipid extraction experiments, as well as the remainder of the data once a singular aggregate state is assumed (Supplementary Figure 23). Physically, the observations by SPR within the model are consistent with all previous observations. The aggregate formation likely accounts for the irreversible baseline shift due to the permanent addition of oligomer to the membrane surface and dramatic structural changes. The intermediate step corresponds to molecular- or macromolecular-binding states that are revealed to be rapid and reversible by the SPR.

Across concentrations, the SPR model fitted and suggested the irreversible aggregate (OL*) to have a concentration threshold similar to the MIC. Model fits were obtained for all oligomer concentrations (Supplementary Figures 23–25, Supplementary Table 2). Within the model, any parameter responsible for directing antimicrobial activity should demonstrate favor for the Para at concentrations below the MIC_Meta_, while showing similarity near the MIC_Meta_. Within the fitted parameters, three matched this criteria: K1, the equilibrium constant for molecular binding states; OL*, the population of irreversible surface aggregates; and to some extent, Loss(OL*), which was lipid particle loss from the aggregate. K1 showed a favorable differential for the Para and occurred at a fast time-scale (seconds), typical of electrostatic associations (Supplementary Figure 26–27). However, the time-scale of the oligomer antimicrobial action was much longer (hours) as previously reported^23^. Thus, K1 was likely not the parameter responsible for biological activity. The population of aggregates (OL*) showed a good correlation with the differential in biological activity and has a slower time-scale to match biological experiment. Lipid particle evolution from the aggregate Loss(OL*) showed some concentration dependence as well (Supplementary Figure 28). Thus, the SPR modeling corroborated the importance of the aggregate formation, as it relates to the biological activity of these isoamphipathic oligomers.

## Discussion

This work has focused on the development of new experimental parameters for membrane-targeting antimicrobials, outside of the known properties of cationic charge and hydrophobicity. OligoTEAs have enabled the synthesis of isoamphipathic oligomers to specifically probe additional parameters in membrane disruption. With extensively similar physicochemical properties, the Meta and Para also displayed similar solution-phase structure and dynamics, indicating they approached the bacterial membrane in similar manners. However, at the membrane surface, the action of the Meta and Para was distinguishable and clear, as shown by the PI and diSC_3_5 assays.

Fluorescence microscopy visualized the formation of a multimeric lipid aggregate, a differential of the two isoamphipathic oligomers. The kinetics, size, and efficiency of aggregate formation favor the Para, potentially connecting it to the biological differential in MIC. FRAP allowed the general visualization of molecular-scale diffusivity, indicating reversible molecular-scale binding. The oligomer-membrane disruption was observed across multiple length-scales by time-lapse fluorescence microscopy, quantification of lipid extraction, and FRAP, all culminating into a kinetic framework that was tested and supported by SPR. The aggregate population (OL*) correlated with the observed behavior in the microscopy and the biological activity across concentrations.

Much research has focused on nano- and/or molecular-scale including models including the barrel-stave pore model, toroidal pore model, carpet model, and others by use of transmission electron microscopy, atomic force microscopy, and X-ray scattering. More recent work has highlighted the dynamic nature of some these states, possibly allowing for reversibility as seen in the FRAP data herein^5,7,53–55^. Other investigations into the mode of action of AMPs and their mimetics have also visualized dramatic, micrometer-scale membrane changes by electron microscopy and confocal microscopy^25,56–58^. These techniques frequently show morphological changes indicative of pore formation, but lack quantitative description this work has demonstrated. With SPR, AMPs and AMP mimetics have demonstrated elevated baselines after injection, meaning aggregate formation or a similar process could be shared between the Meta and Para with others scaffolds^7,35,51,59^. Outside the scope of this work, the robust appearance of this multimeric lipid aggregate on supported bacterial-derived membranes^38^ will be explored in future work with other AMP and AMP mimetic scaffolds to see whether similar behaviors are observed.

In this work, lipid aggregate formation is implicated in the mechanism of action of membrane-disrupting materials. However, it is not immediately clear how aggregate formation induces antimicrobial action. The Para produces both a greater number and smaller size of aggregates and either parameter (size or quantity) could be responsible. A smaller aggregate indicates efficient aggregate formation, requiring a lower oligomer-to-lipid ratio, analogous to the peptide-to-lipid ratio^6^. However, It is also possible that the oligomers have similar ratios, but the Para provides more favorable kinetics for irreversible aggregate formation. Either way, the states of future interest lay somewhere between oligomer binding (OL) and aggregate formation (OL*). In this work, SPR could not distinguish additional states without introducing redundancy in the model. Investigating deeper into these quick transitions at the molecular and nanoscale states remains challenging. With respect to these oligomers, the challenge also included connecting the molecular conformation to the nano- and micrometer scale. With requirements of high time- and length-scale resolution, we expect that further investigation with molecular simulation will provide answers when focused on the complexation of the oligomer with lipids and subsequent multiplex formation.

In the quest to develop new antibiotics in the face of antimicrobial resistance, we hope this work underlines multimeric lipid aggregation as a new biophysical parameter for further design and optimization of membrane-targeting antimicrobials. Advances in chemistry continue to enable the development of sequence-defined polymers and oligomers designed to ask specific fundamental questions about the impact of sequence, structure, conformation, and composition. Although not directly observed in this work, a specific mode of antimicrobial action, pore formation, has been seen to be sensitive to differences as small as chirality in AMP mimetic structure, necessitating precise chemical control^54^. Here, OligoTEAs provided isoamphipathic oligomers, demonstrating the same physicochemical properties as well as the same solution-phase structure and dynamics, but a unique differential allowing the demonstration of this new biophysical property.

## Methods

### Chemicals and reagents

Chemicals were purchased from Sigma Aldrich, Alfa Aesar, or Acros Organics, or Ark Pharm. Fluorous tag and fluorous silica were purchased from Boron Specialties. Strains of *B. subtilis* were kindly donated by the Helman Research Group (Microbiology, Cornell University). MRSA (ATCC 33591), Vancomycin-resistant *Enterococcus faecium* (VRE; ATCC 700221), and *Staphylococcus epidermidis* (ATCC 14990) were obtained from ATCC. Human red blood cells were purchased from Innovative Research (Novi, MI).

### General method for oligoTEA assembly

Oligomers were prepared as described starting with a C_8_F_17_ fluorous tag^40,60,61^. The oligomer was iteratively assembled by reacting fluorous-bound allyl groups with dithiol monomers via a thiolene reaction to produce a mono-substituted thiol to be reacted in a thiol-Michael addition with an allyl acrylamide monomer. After thiol-Michael addition, the fluorous olefin was regenerated with each cycle. Fluorous solid-phase extraction after each reaction purified and removed excess reagent. The fluorous product was retained selectively and purified on fluorous silica (~ 40 mg/mg fluorous tag) during a fluorophobic wash of 20–30% Water in MeOH (0.4 mL/mg fluorous tag) and eluted with MeOH (0.2 mL/mg fluorous tag). Oligomers were dried by nitrogen or by vacuum centrifuge (both RT). OligoTEAs were cleaved with 100% trifluoroacetic acid, HPLC purified, and verified by LCMS and/or ^1^H NMR where appropriate.

### MIC assay

A single colony was selected and grown overnight in broth media (*B. subtilis*, Luria Broth; *S. epidermidis*, MRSA, VRE, Tryptic Soy Broth). A subculture was then grown to mid-exponential phase (37 °C) and OD600 was measured and diluted to 0.001 with designated media. OligoTEA or antibiotic and bacterial suspension were combined (5:95 v/v%) in a 96-well plate and incubated (37 °C) with agitation to the final concentration reported by serial dilution. After 14 h, the OD600 was measured. Data were normalized to the media blank (0) and solvent control (1). The MIC was recorded as the lowest (minimum) concentration required to kill all visible bacteria cells (e.g., see Supplementary Figure 2).

### Pulse-field gradient nuclear magnetic spectroscopy

Measurements were performed with a Varian Unity INOVA 600 MHz spectrometer with a Varian 600 triple resonance XYZ PFG (HCN) inverted probe. The 90° pulse angle was optimized. Diffusion measurements were accomplished using the double-stimulated echo convection compensated sequence^62^ using 3 mm tubes and 20 LPM of gas flow to diminish convection^63^. Measurements were completed with an acquisition time of 1.7 s, 8 steady-state pulses, diffusion gradient length of 2.0 ms, 0.0 ms of off-center delay (del2), 0.00 unbalancing factor, alternating gradient pulse sign, and diffusion delay of 120 ms. Scout measurements helped determine the gradient stabilization delay (1.0–2.5 ms) to minimize eddy currents. A standard (99.9% D_2_O) was run to calibrate the probe (gcal) by the observed diffusion coefficient of HDO for each temperature as described by a Speedy–Angell power law fits^64^ of Longsworth’s data^65^.

### Double electron–electron resonance EPR

DEER ESR measurements were completed at the National Biomedical Center for Advanced ESR Technology (ACERT) Center at Cornell University. Di-spin labeled oligoTEAs were reduced using 1 N aqueous ammonia for 1–2 h at 50–500uM at RT and dialyzed against ultrapure water using a 100–500 MWCO Micro-Float-A-Lyzer (Spectrum Labs) and monitored by a calibrated Accumet (Cat 13-620-165) conductivity meter. All samples were prepared at 50 μM and vitrified to 70 K rapidly from RT. A working frequency of 17.3 GHz with a 30 G magnetic component in a rotating reference frame was sufficient for distances of 10 Å or longer. Samples were measured by 4-pulse sequence DEER for 1 μs. Time domain data were processed in MATLAB to determine sensitivity to baseline fitting. Then, distance distributions were calculated by Tikhonov regularization based on the L-curve method (*α* ~ 2–7) using MATLAB scripts from the ACERT website (acert.cornell.edu).

### General method for X-ray scattering

All samples were centrifuged at 14,000 RPM for 10 min. SAXS data were collected at Cornell High Energy Synchrotron Source (CHESS) beamline G1 at ~ 12.68 keV (0.98 Å) at 5.5 × 10^9^ photons per second. The X-ray beam was collimated to 100 μm^2^ diameter and centered on a sample cell with 1.5 mm path length and 5 μm thick glass walls (Nippon Electric Glass America, Schaumburg, IL). The sample cell and full 1.5 m X-ray flight path, including beamstop, were kept in vacuo (1 × 10^−3^ torr) to eliminate air scatter. Sample plugs of 30 μL were delivered to the capillary. To reduce radiation damage, sample plugs were oscillated in the X-ray beam using a peristaltic pump (Ismatec, Cole-Parmer GmbH, Germany). Images were collected on a dual Pilatus 100K-S detector system (Dectris, Baden, Switzerland) for small- and wide-angle scattering observation with 20–80 sequential 1 s exposures being used to assess possible radiation damage. Sample and buffer solutions were normalized to equivalent exposure before subtraction using beamstop photodiode counts. Sample-to-detector distance was calibrated using silver behenate powder (The Gem Dugout, State College, PA). Images were averaged to profiles and buffer subtracted using the BioXTAS RAW software (1.4.0), which includes a statistical check for radiation damage during reduction. Slight buffer mismatch was observed, evident most in the WAXS region. The offset sample signals were seen to decay fully in the WAXS region at approximately *q* = 0.6–0.7 and data were thus scaled appropriately at *q* = 0.7 (scale factors were ~ 3% from original). The useful *q*-space range (4*π*Sin*θ*/*λ* with 2*θ* being the scattering angle) was generally from *q*min = 0.01 Å^−1^ to *q*max = 0.7 Å^−1^.

### PI membrane permeabilization assay

A single colony was cultured overnight and then subcultured and incubated 3 h until the OD600 measured between 0.5 and 0.6. Bacteria were collected, washed, and resuspended in a solution of 5 mM HEPES buffer, 5 mM glucose, and 10 μM PI at pH 7.2. A total of 150 μL of bacteria solution was added to each well of a black 96-well plate. Fluorescence measurements were taken at 535 nm excitation/617 nm emission on a TECAN Infinite M1000 PRO Microplate reader (Männdorf, Switzerland) for 2 min. Oligomer stock solutions in water were added to give a final concentration of 15 μM and fluorescence measurements were taken for an additional 20 min.

### DiSC_3_5 membrane depolarization assay

A single colony was cultured overnight and then subcultured. The bacteria were collected and washed twice with HEPES buffer. EDTA solution (0.5 M, pH 7.4) was added for a final concentration of 0.2 mM EDTA and diSC_3_5 was added for a concentration of 0.4 µM. The solution was incubated 30 min, before adding 100 mM KCl and incubating 1 min. This bacterial solution was added to oligomer stocks in a black 96-well plate and the fluorescence intensity (610 nm excitation, 660 nm emission) was recorded for 60 min. The data were analyzed by subtracting the baseline (water) from the sample intensity at 60 min.

### Fluorescence microscopy and FRAP

To form planar SLBs, polydimethylsiloxane (PDMS) wells of ~ 1 cm diameter were attached to piranha-washed glass slides (70% sulfuric acid, 30% hydrogen peroxide). PDMS consisted of 10:1 elastomer:cross-linker mixture of Sylgard 184 (Robert McKeown Company). Wells were coated with 100 μL of PLL (0.1%wt/vol in water, Sigma P8920) for 30 min (RT), then washed with PBS pH 6.8. SUVs were labeled with 0.05–0.1 mol% Octadecyl Rhodamine B or Texas Red^TM^ DHPE (Molecular Probes). The labeling amount was kept low to prevent surface quenching. G25 spin column (GE Healthcare) removed excess fluorophore. Labeled vesicles were added to well and incubated for 10 min to rupture and form a bilayer. The well was gently rinsed with PBS to wash away excess vesicles. Scratches were made on the bilayer to aid in determining the focus. Imaging was performed with a Zeiss Axio Observer.Z1 microscope with α Plan-Apochromat × 20 objective. FRAP measured lipid diffusion after photobleaching^66^ after oligomer exposure (1 h) and after washing (5–10 mL PBS). A ~ 20 μm diameter spot in bilayer was photobleached by 150 mW 561 nm optically pumped semiconductor laser (Coherent, Inc.) for 100 ms. The recovery of was recorded and fit in comparison with the background following the method reported by Soumpasis^67^. The diffusion coefficient was calculated using the equation, *D* = *w*^2^/4*t*_1/2_, where the full width half-maximum *w* of the Gaussian profile is used for calculation.

### Surface plasmon resonance

SPR was completed using a Biacore 3000 with an L1 Chip at 25 °C modified slightly from the manufacturer’s protocol. Before use, desorb, sanitize, and an overnight wash with ultrapure water were completed. PBS (1 ×, pH 6.8) was used throughout all runs and solutions. The chip was conditioned with 7 μL 40 mM octyl-β-glucopyranoside (Alfa Aesar) at 10 μL/min at the start and end of each run. Additional manufacturer recommended washes were used. SUV capture was done for 10 min (5 μL/min) to ensure surface coverage. The following control runs were performed as follows: (i) 1 min pulses of 10 mM NaOH showed little response to indicate minimal formation of multilayers, (ii) injections of the oligomer alone showed no response, and (iii) injections of 0.1 mg/mL bovine serum albumin showed little to no response. At an equilibrated flow of 30 μL/min, samples were injected (kinject) for 5 min and dissociated for 6 min with PBS.

### Supplementary Information

Synthesis, additional detail for methods, NMR, and LCMS verification of oligoTEA structures are provided. ^1^H NMR and LCMS spectra are shown in Supplementary Figures 29–40. Data, methods, and analysis methods from the solution-phase characterization are also provided in the supplementary materials. Fluorescence microscopy movies, ImageJ threshold examples, and other microscopy data are provided. All SPR sensorgrams including parameters from all fits and error are provided.

### Code availability

Custom code used in MATLAB to fit the model to the experimental SPR data as described is available in the Supplementary Information.

## Electronic supplementary material

Supplementary File

Description of Additional Supplementary Files

Supplementary Movie 1

Supplementary Movie 2

Supplementary Movie 3

## Data Availability

The authors declare that all data not present within the supplementary information are available from the corresponding author upon reasonable request.
