# Supplementary material for: Antibacterial isoamphipathic oligomers highlight the importance of multimeric lipid aggregation for antibacterial potency
Source: Commun Biol. 2018 Dec 7;1:220. doi: 10.1038/s42003-018-0230-4 (PMC6286309; doi:10.1038/s42003-018-0230-4)
Supplement: Supplementary file 1 — Supplementary File [file 42003_2018_230_MOESM1_ESM.pdf]

Antibacterial isoamphipathic oligomers highlight the importance of multimeric lipid aggregation for antibacterial potency

Robert Frederick Smith School of Chemical & Biomolecular Engineering,
120 Olin Hall, Cornell University, Ithaca, NY 14853 USA

Address materials and correspondence to Christopher A. Alabi (caa238@cornell.edu)

SUPPLEMENTARY INFORMATION

Supplementary Figures

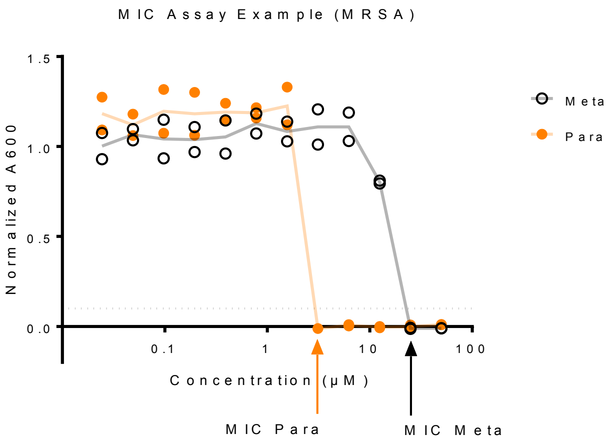

Supplementary Figure 1 | Example of MIC assay to demonstrate MIC definition. The minimum inhibitory concentration is defined as the concentration at which all bacteria cells (>90%) are killed; as indicated by the arrows for the Meta and Para. This has also been termed a MIC_{90}.

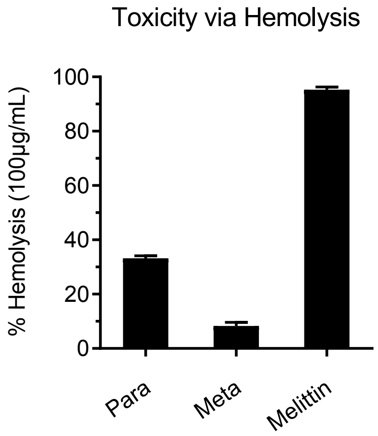

Supplementary Figure 2 | Hemolysis (Red blood cell) assay of Meta and Para. Hemolysis was done at 75 μM with the method above. Melittin included as a positive control of a pore-forming membrane-disrupting agent. Error bar represents the standard deviation *(n=2 biological replicates)*.

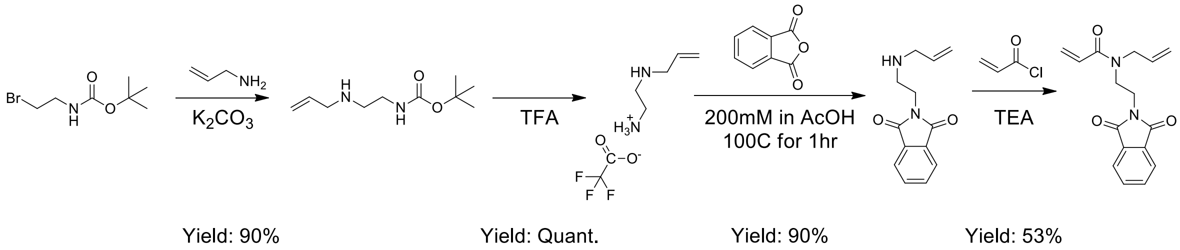

Supplementary Figure 3 | Scheme to synthesize the phthalimide-protected amine N-allyl-N-acrylamide. More description and detail are provided in the Supplementary Methods, under the section on *Spin-labeling the oligomer scaffold*.

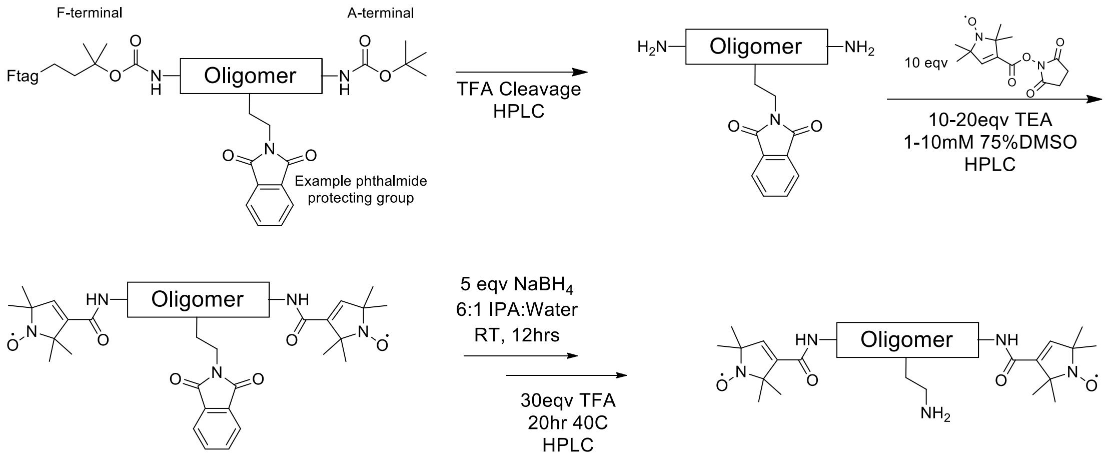

Supplementary Figure 4 | Scheme to produce polyamine dispin-labeled oligothioetheramides. More description and detail are provided in the Supplementary Methods, under the section on *Spin-labeling the oligomer scaffold*.

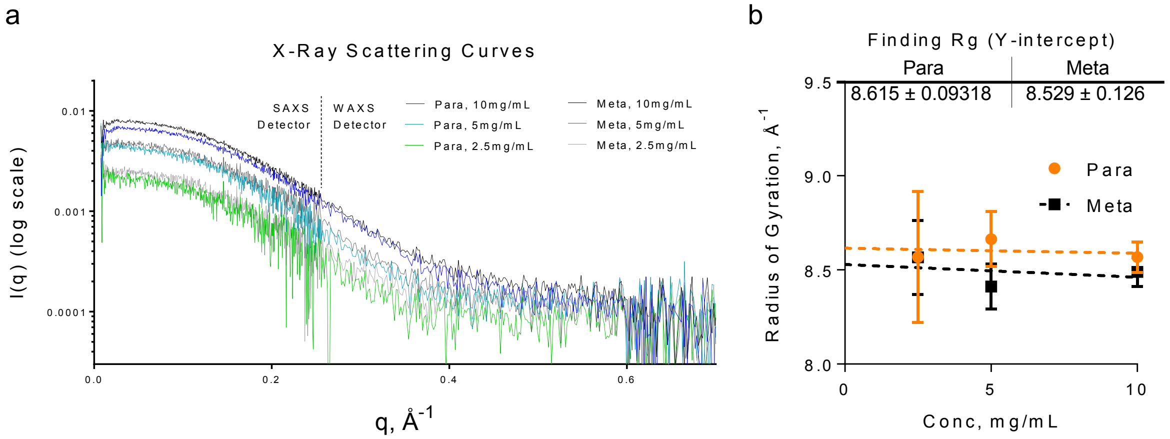

Supplementary Figure 5 | X-ray scattering and radius of gyration (Rg) determination. (a) PBS subtracted x-ray scattering curves were reduced as described above. Signal from the oligomers decays broadly into the wide-angle regime reflecting their small size. **(b)** The radius of gyration was determined by extrapolating to infinite dilution (y-intercept) from the radii at individual concentrations determined by fitting within the Guinier regime (Table S1).

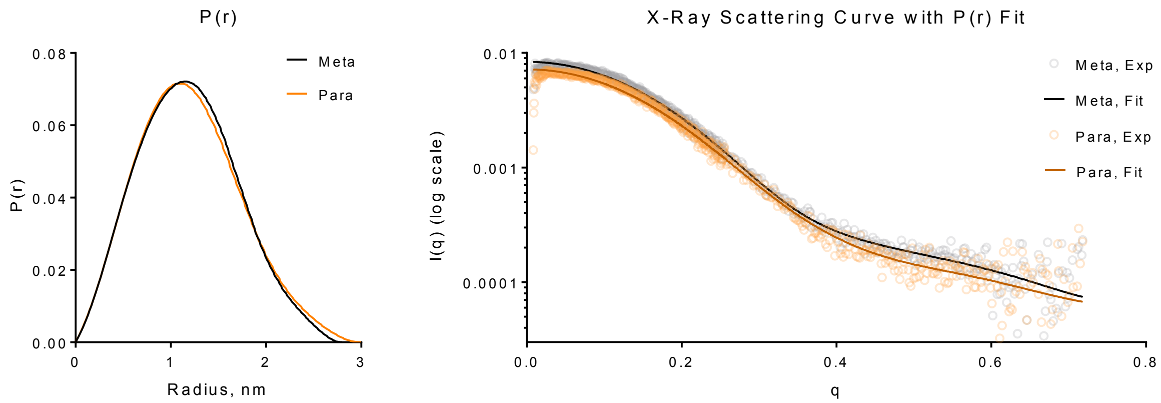

Supplementary Figure 6 | Pair-wise distribution reconstructed from the x-ray scattering profiles. (left) Pair-wise distribution generated by GNOM[13] within BioXTAS RAW.[14] Dmax was calculated to be 2.8nm and 3.0nm for the Meta and Para, respectively, based on DATGNOM. **(right)** Raw scattering data and the fit solution used in the reconstructed pair-wise distribution.

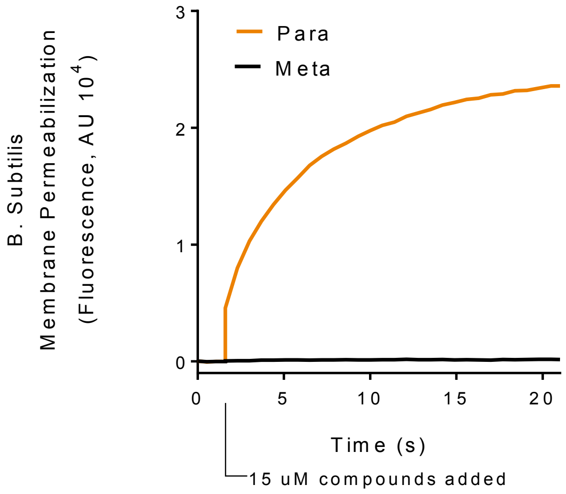

Supplementary Figure 7 | PI assay of Para and Meta oligomers with B. subtilis.

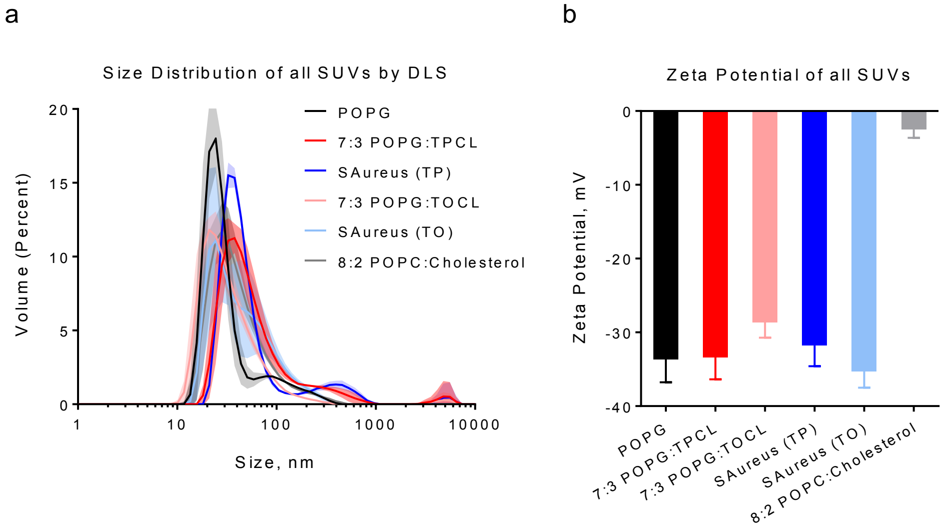

Supplementary Figure 8 | Dynamic light scattering and zeta potential of all SUVs. (a) Volume distribution of all SUVs revealing compositions are comprised of mostly 20-80 nm particles. **(b)** Zeta potential measurements of all SUVs revealing their strong anionic surface charge, with the exception of the mammalian mimetic composition 8:2 POPC:Cholesterol. Even with the addition of neutral lipids, the *S. aureus* mimetic SUVs maintained a strong negative charge. TP and TO refer to the cardiolipin tail lengths explored. TPCL was used because it provided a better quality membrane after inspection by fluorescence microscopy.

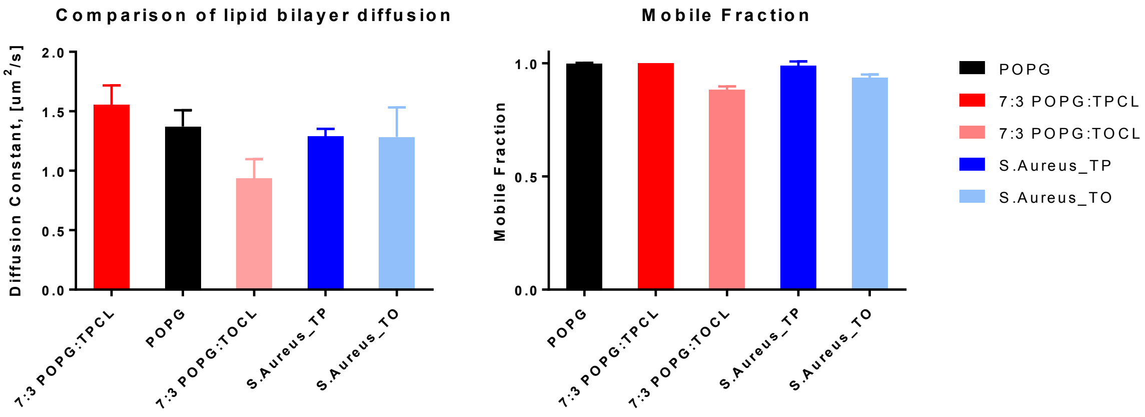

Supplementary Figure 9 | Fluorescence recovery after photobleaching (FRAP) of all mimetic membranes. All membrane mimetics show similar membrane fluidity and diffusion between 1-1.5 μm^2/s and mobile fractions at room temperature (22°C). The diffusivity of these membranes is modestly faster than bacterially isolated membranes from outer membrane vesicles (OMVs), which demonstrate a diffusivity of approximately ~0.5 μm^2/s.[28]

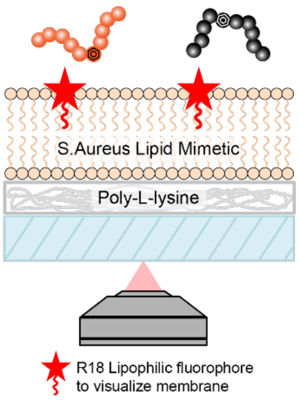

Supplementary Figure 10 | Diagram of fluorescence microscopy set-up. The lipid bilayer is visualized by a lipophilic dye R18 at 0.05-0.1 mol% (rhoadmine with C18 alkyl tail). A poly-L-lysine (Sigma P8920, 150-300k MW) was necessary to enable the formation of lipid bilayers on glass microscope slides due to the SUVs' net negative charge.

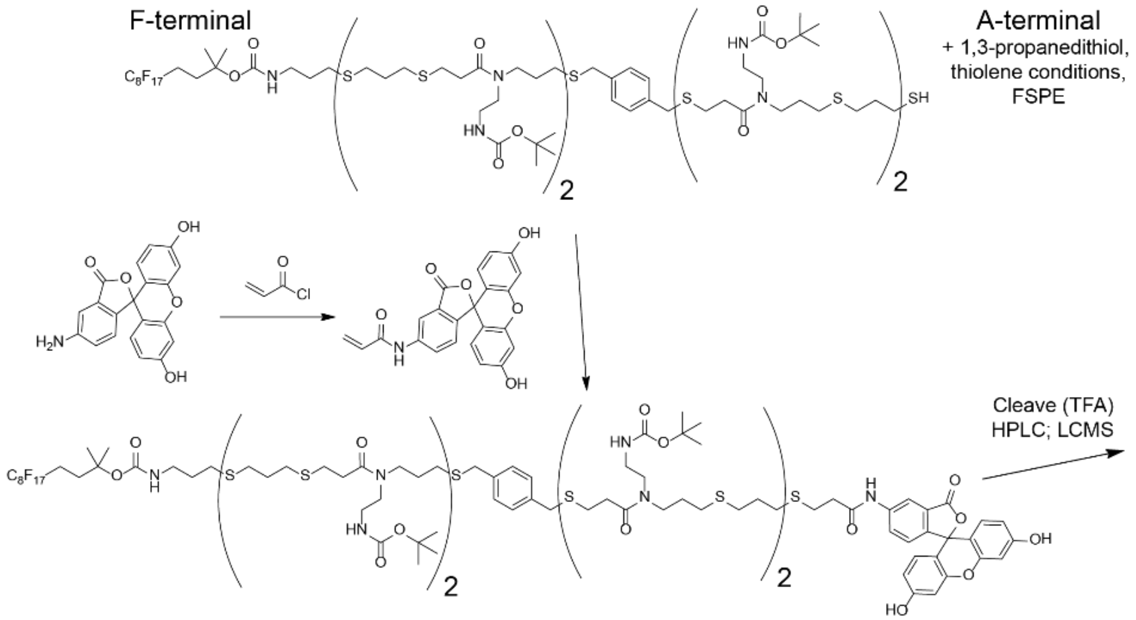

Supplementary Figure 11 | Scheme to fluorescein label the Meta and Para. Before the termination of oligomer assembly, fluorescein acrylamide was conjugated to the terminal thiol of 1,3-propanedithiol by thiol-Michael addition conditions catalyzed by 1,8-Diazabicyclo[5.4.0]undec-7-ene (DBU) instead of DMPP, as DMPP did not lead to efficient reaction conversion.

Fluorescein acrylamide was prepared from the treatment of fluorescein amine (227 mg, 0.606mmol, Acros Organics) with 2eqv of N-methylmorpholine (pKa 7.4, to minimize di-addition product) in 100mM THF, with 1.1eqv of acryloyl chloride added dropwise while stirring at 0C for 6 hours. The crude mixture was extracted into ethyl acetate and washed with 1M HCl twice and brine. Flash chromatography 0-20% MeOH in DCM eluted the product at 5% MeOH (Yield: 40 mol%). Once the fluorescein acrylamide was conjugated to the fluorous terminal, FSPE was performed, followed by 100% TFA cleavage, HPLC, and verification by LCMS.

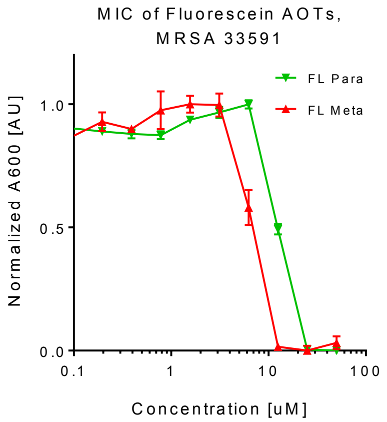

Supplementary Figure 12 | Minimum inhibitory concentration (MIC) of fluorescein olgiomers. The fluorescein labeling of the Meta and Para decreases their potency against MRSA 33591 by about 10-fold, but should hopefully serve to similarly localize and/or function with the unlabeled oligomers.

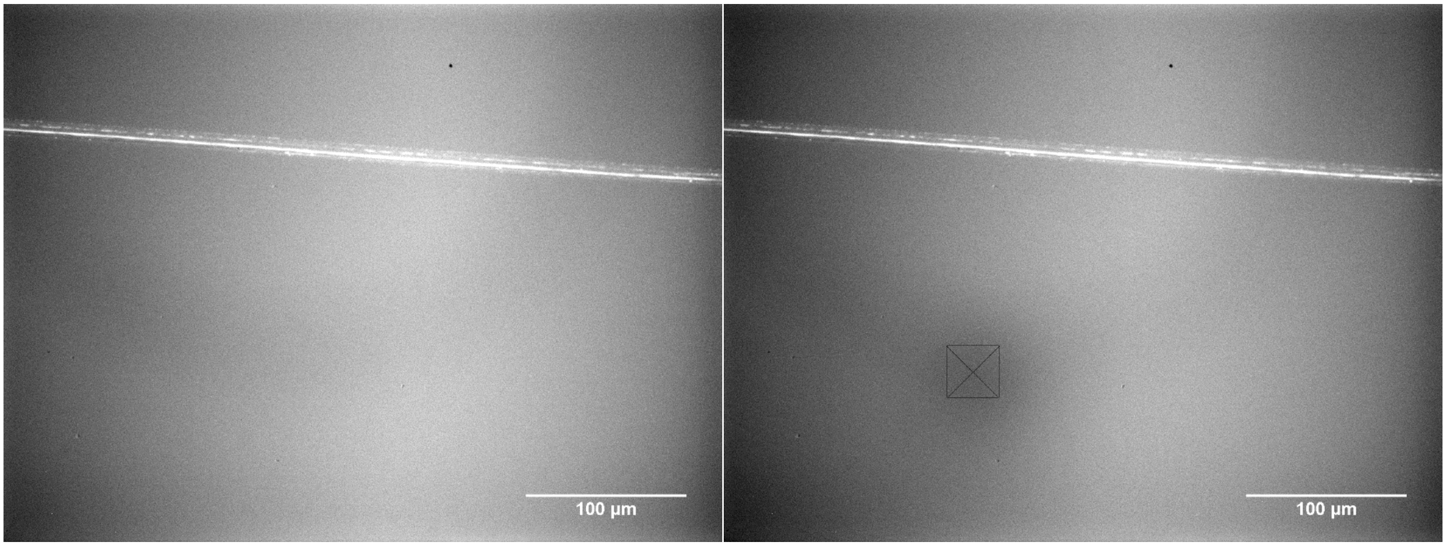

Supplementary Figure 13 | Confirmation of lipid extraction by Triton-X detergent. There was little remaining signal, but these images were captured by maximizing the fluorescence excitation and using a long exposure time. The scratch was created while the bilayer was present (before removal) to find the focal plane. Microscopy slide was then treated with 5mM Triton-X to remove the R18-labeled lipid bilayer. **(left)** The bilayer removal was verified visually **(right)** and also by the lack of a photo-bleached spot by the FRAP laser (100ms exposure, which was located in the black crosshairs).

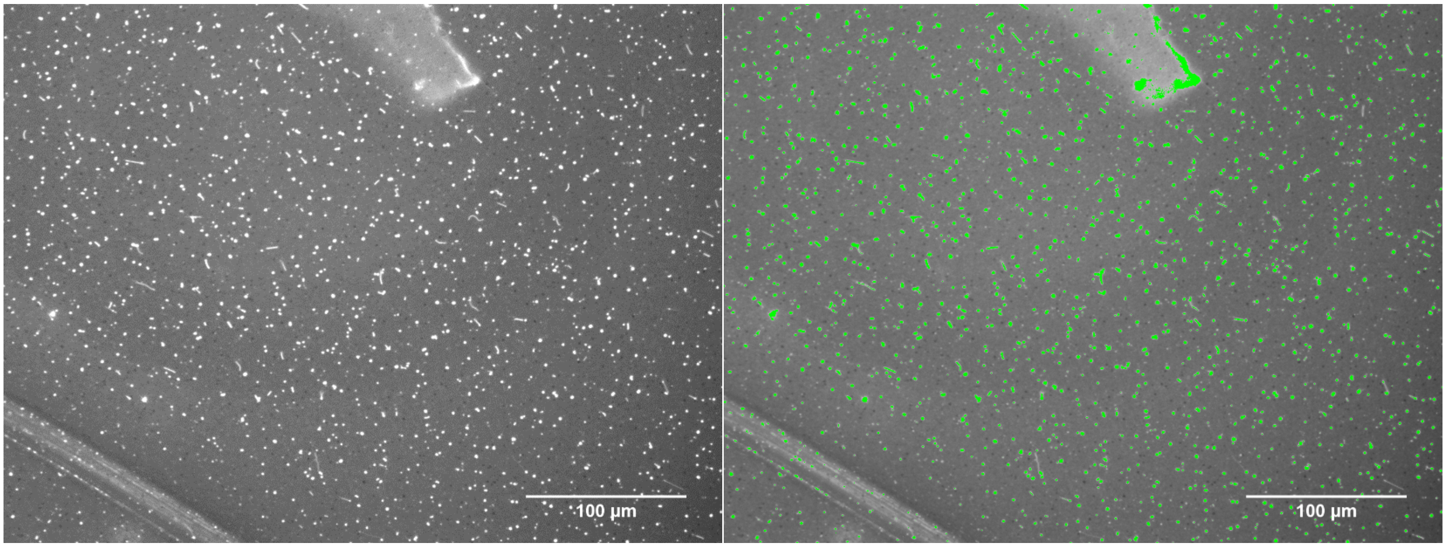

Supplementary Figure 14 | ImageJ thresholding of aggregates after 5μM Meta exposure. (left) The original microscopy image with aggregates (white dots) that form after treatment of the *S. aureus* mimetic membrane with 5μM Meta for 10 minutes. The scratch in the lower left corner was created to aid focus on the lipid bilayer. **(right)** ImageJ composite image of thresholding, highlighting green where aggregates are located. Some minor undercounting of small particles is observed. Particle thresholds were then processed with ImageJ using Particle Analysis to report the number and size of the aggregates.

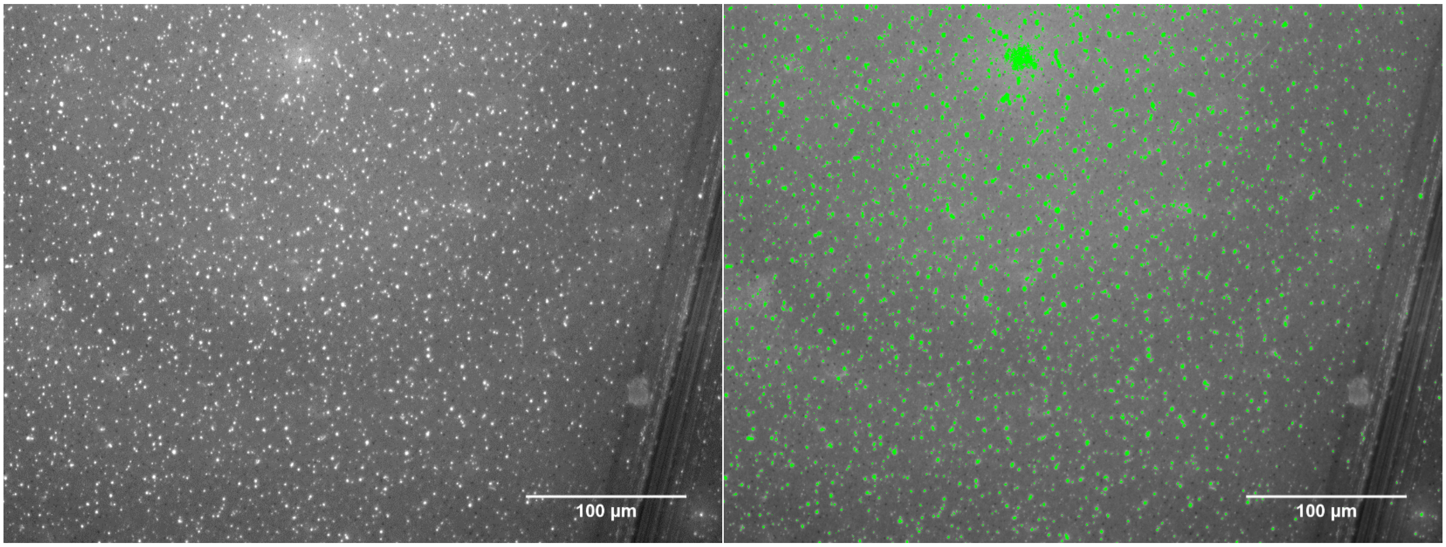

Supplementary Figure 15 | ImageJ thresholding of aggregates after 5μM Para exposure. (left) The original microscopy image with aggregates (white dots) that form after treatment of the *S. aureus* mimetic membrane with 5μM Para for 10 minutes. The scratch in the lower left corner was created to aid focus on the lipid bilayer. **(right)** ImageJ composite image of thresholding, highlighting green where aggregates are located. Some minor undercounting of small particles is observed. Particle thresholds were then processed with ImageJ using Particle Analysis to report the number and size of the aggregates.

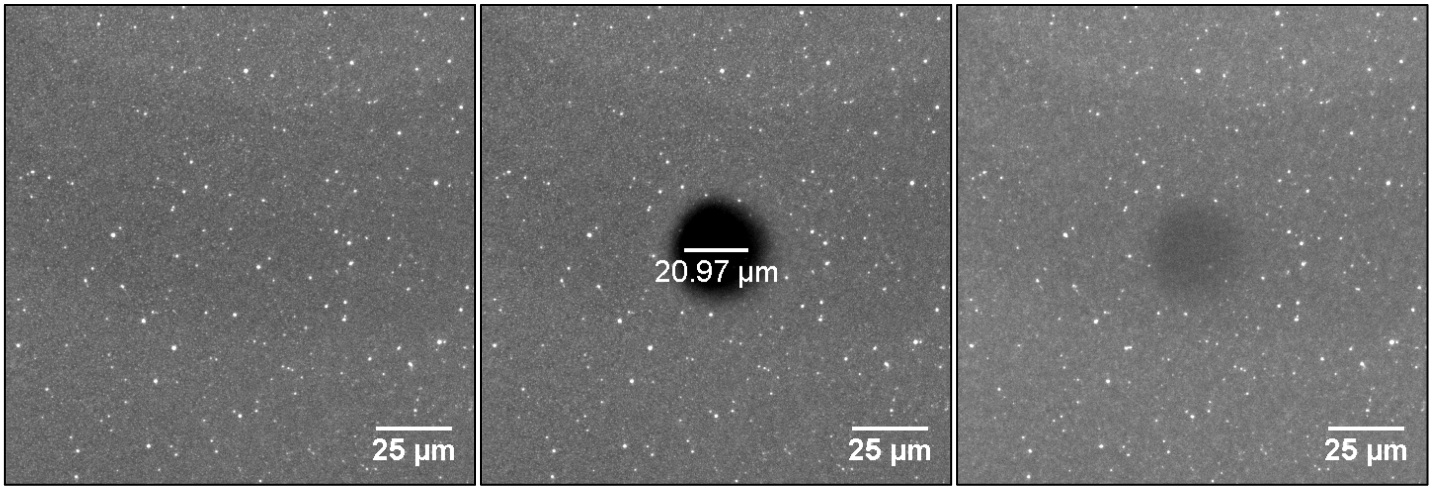

Supplementary Figure 16 | Size of the FRAP photobleached spot. (left) An *S. aureus* mimetic membrane on a PLL substrate was treated with 5µM Para for 1 hour and washed with PBS. (t=0) **(middle)** A ~20 µm diameter spot in bilayer was photo-bleached by 150 mW 561 nm optically pumped semiconductor laser (Coherent, Inc.) for 100 ms **(right)** The recovered FRAP spot 30 minutes later showing that the aggregates are immobilized and they do not recover their spot fluorescence with a small immobile fraction.

Calculation to estimate the aggregate area percentage of the FRAP laser spot. At this concentration (Figure S16), approximately 1500 aggregates were counted in the full viewing area of 450 x 335 µm, meaning the aggregate density is on average 1e-2 aggregates per μm^2. Thus in the ~315μm^2 FRAP spot, ~3 aggregates will be present with variation. In this case, five aggregates were photo-bleached. Assuming each aggregate averages approximately 2 μm^2 in area, the average area of aggregates in the FRAP laser spot would be a total of 6 μm^2, which is approximately 2% of the FRAP spot area. For the maximal aggregate count observed in this study of ~3000 aggregates (at 10uM), that would double this estimation such that the aggregates would be approximately 4% of the FRAP laser spot size.

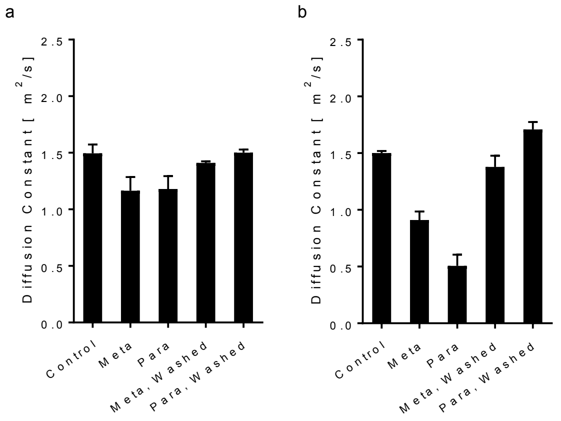

Supplementary Figure 17: FRAP experiment revealing reversible decrease in membrane diffusivity. SLBs were treated and equilibrated with **(a)** 2.5 and **(b)** 5 µM oligomer to show reversible binding of a molecular, non-aggregate state.

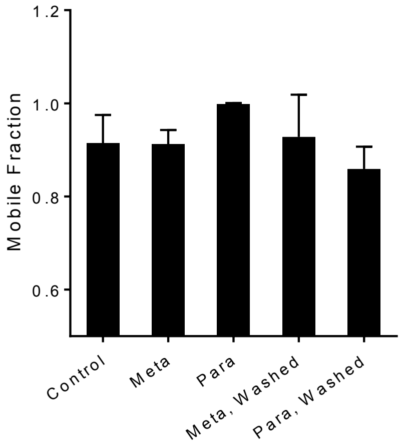

Supplementary Figure 18 | Mobile fraction from FRAP measurements in main text. A small immobile fraction is present as shown by the FRAP as described in Figure 4g.

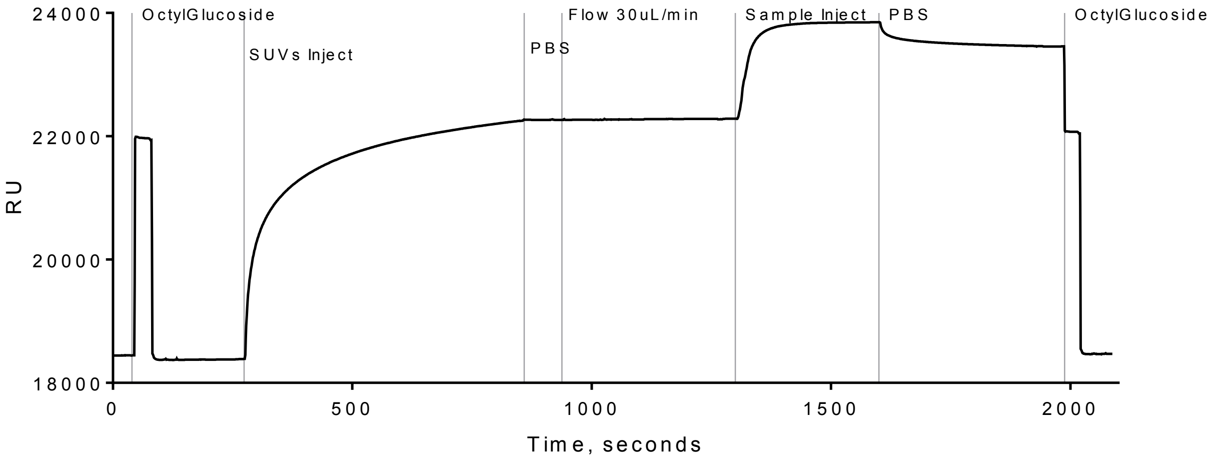

Supplementary Figure 19 | Full sensorgram example of SPR run. 5uM Meta on a *S. aureus* captured mimetic membrane.

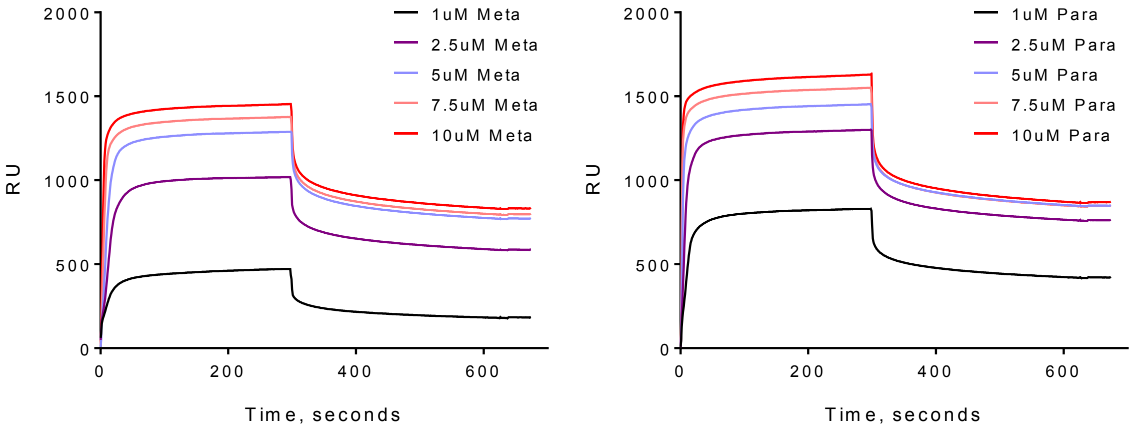

Supplementary Figure 20 | SPR sensorgrams of the Meta and Para on *S. aureus* lipid mimetic. Per literature search and discussion above, the *S. aureus* lipid mimetic composition was 4:5:11 neutral lipids (18:1 DG): TPCL: POPG.

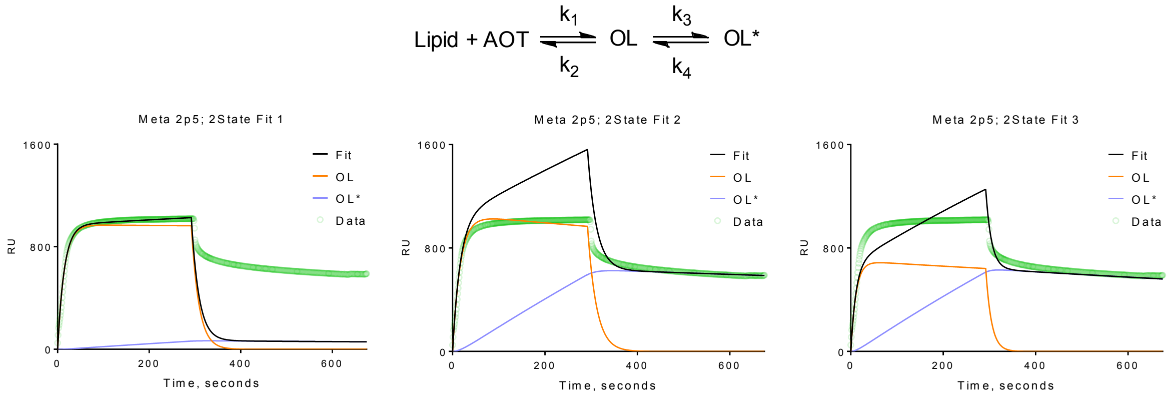

Supplementary Figure 21 | Failure of two-state model without loss to fit SPR sensorgrams. The attempts to fit the data of the 2.5μM Meta with the 2-State model shown were unsuccessful. Fits shown demonstrate that the model could fit the association or the dissociation phase, but not both simultaneously.

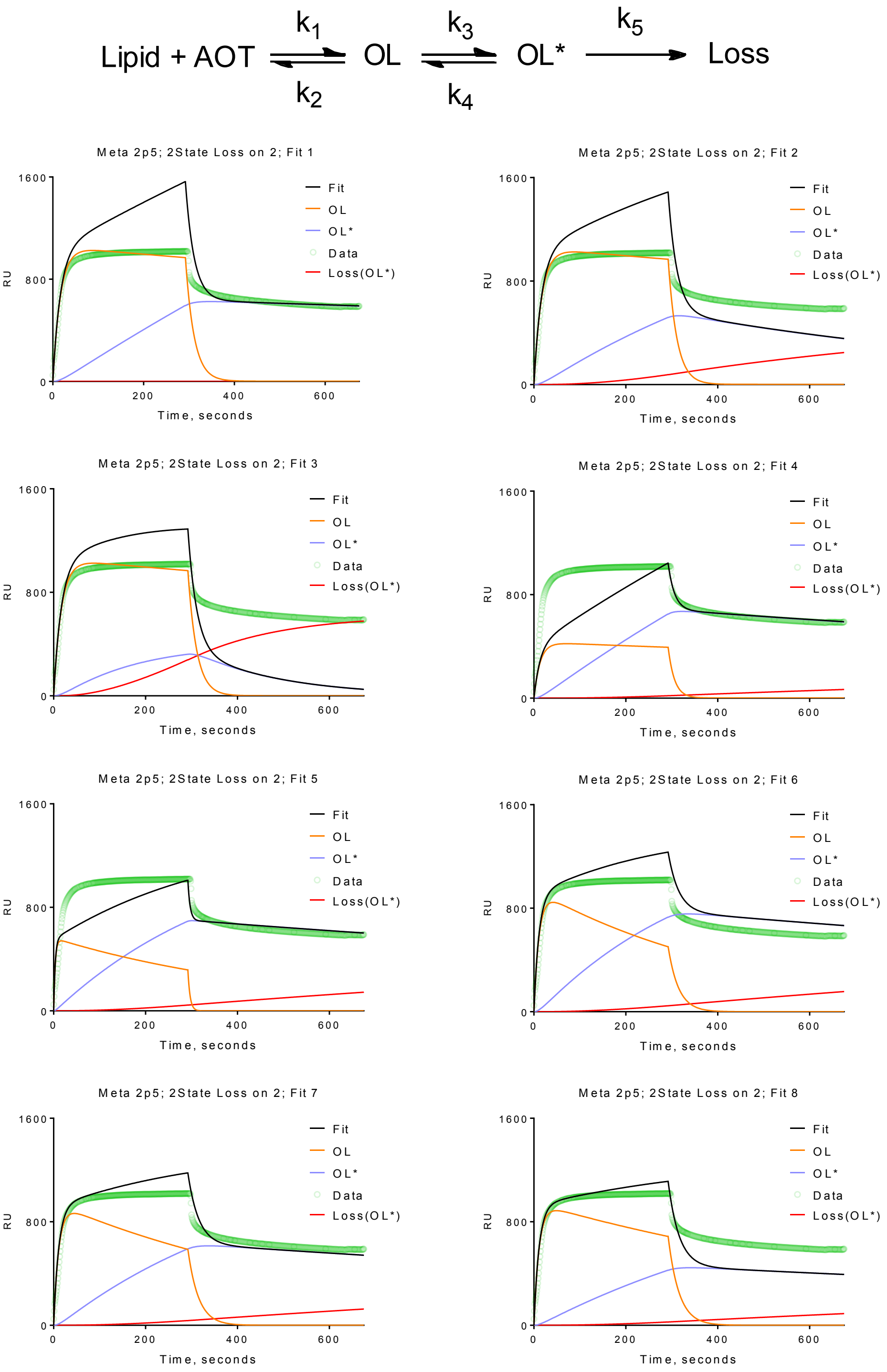

Supplementary Figure 22 | Failure of two-state model with loss to fit SPR sensorgrams. Several attempts to make the 2 State Model with Loss on 2 fit, as it is the prevailing model within literature. However, one can observe how this model can fit multiple sensorgrams seen in other literature. For this observed data, the model could not converge, likely because of either a) the lack of an upward slope in the association phase or b) the substantially elevated baseline in the dissociation phase.

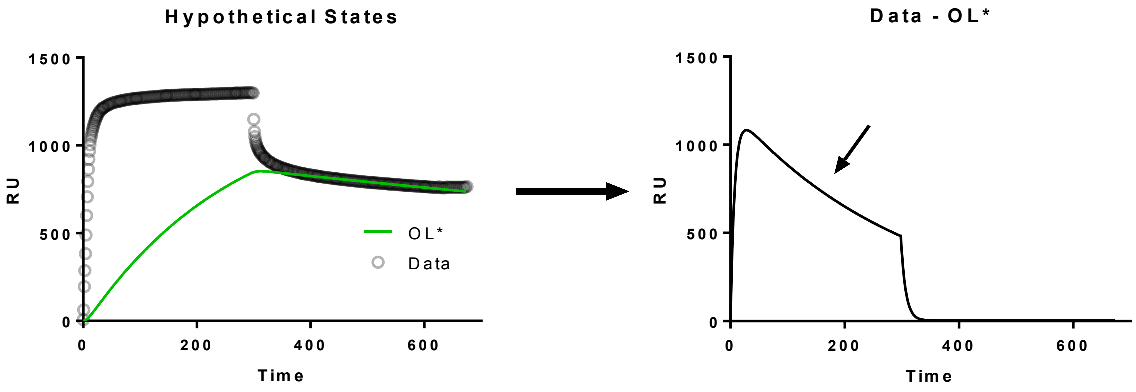

Supplementary Figure 23 | Evidence for loss on intermediate step from assumption of one irreversible state. In assuming irreversible aggregation (OL*) to be responsible for the elevated baseline during dissociation, the data remaining from that assumption shows a curve that would be challenging for fitting with the two state model with loss on the second step. In particulate, the curvature of downward slope (see arrow), would be difficult to fit.

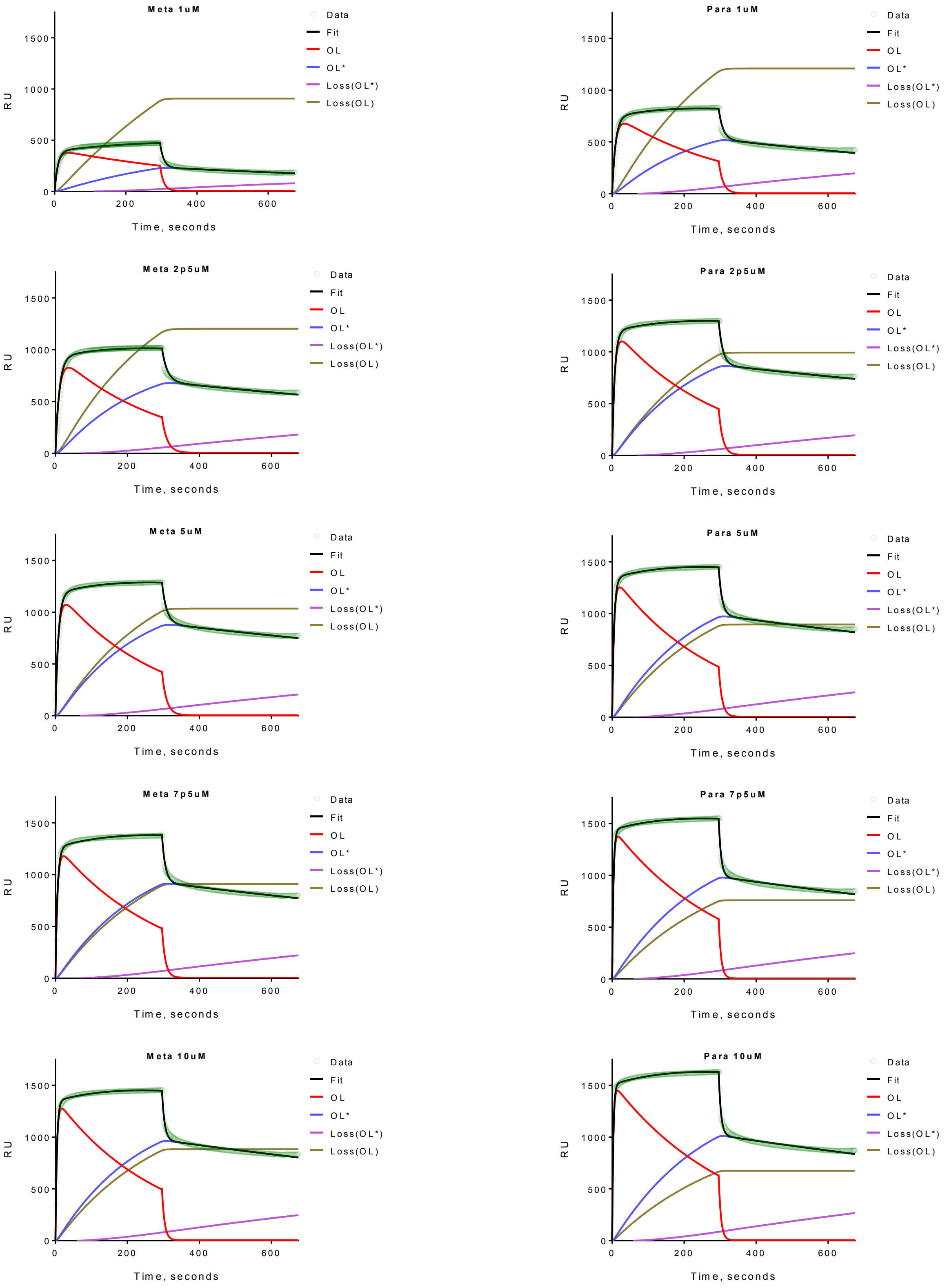

Supplementary Figure 24 | All model fits of SPR data on *S. aureus* mimetic membranes.

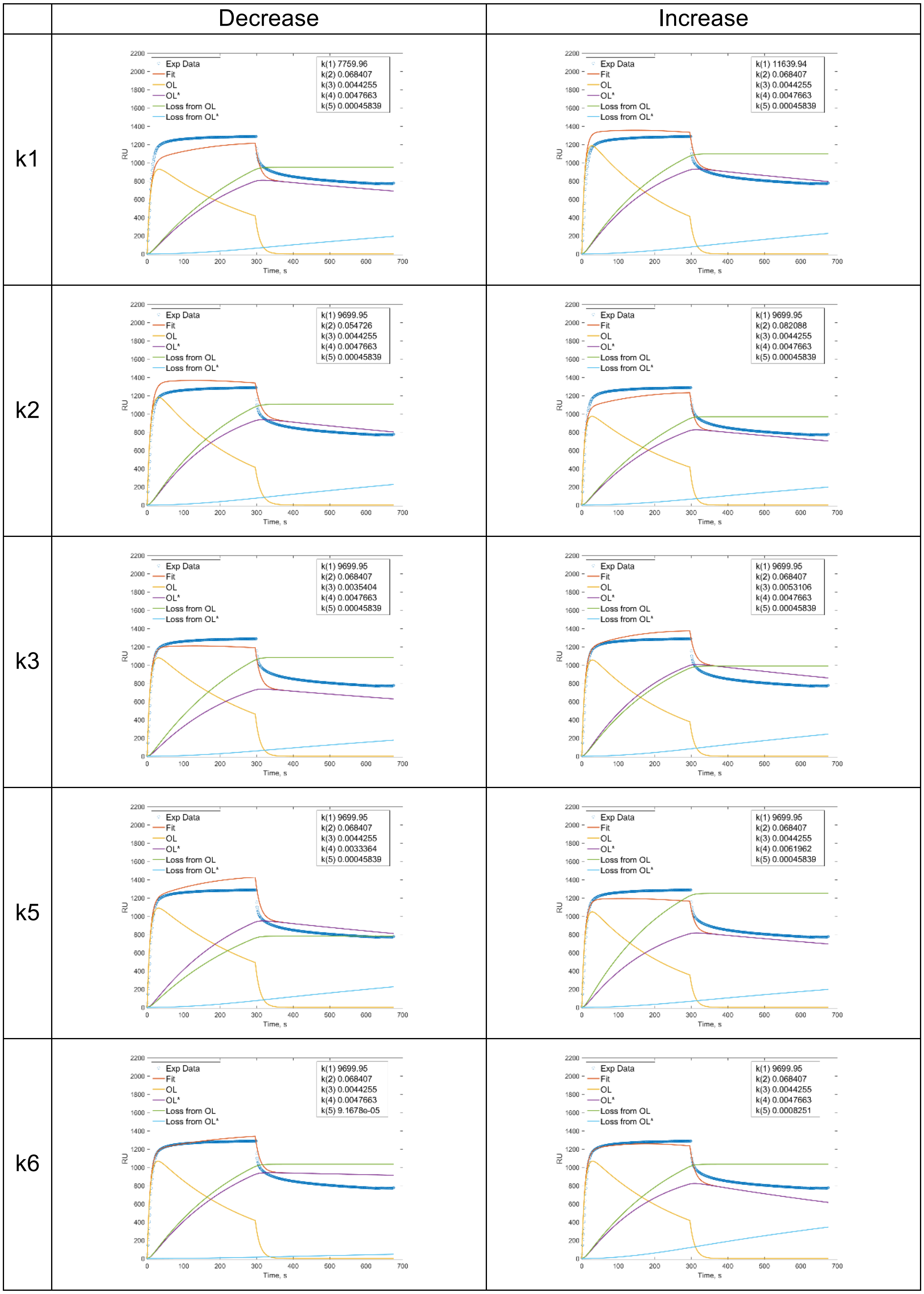

Supplementary Figure 25 | Model behavior demonstrated by varying all parameters on 5μM Meta dataset. Provided to aid the reader in how the model behaves based on its parameter set.

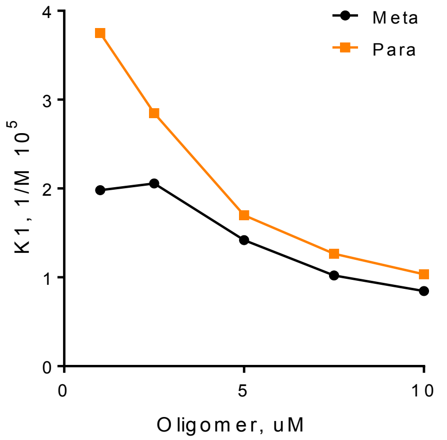

Supplementary Figure 26 | K1 versus oligomer concentration showing a concentration threshold. The equilibrium constant K1 does show the desired concentration trend for a parameter that trends close to the biological threshold (i.e. MIC). Specifically, the Meta K1 does not continue increasing as the oligomer concentration decreases. Physically, it is hard to interpret K1 because it is not simply "binding," as it does encompass several physical states as understood in literature (e.g. binding, tighter binding, insertion, molecular aggregation)[30]. Therefore, this data shows there is perhaps a limit to the cooperativity of this process (i.e. the equilibrium decreases as the concentration increases).

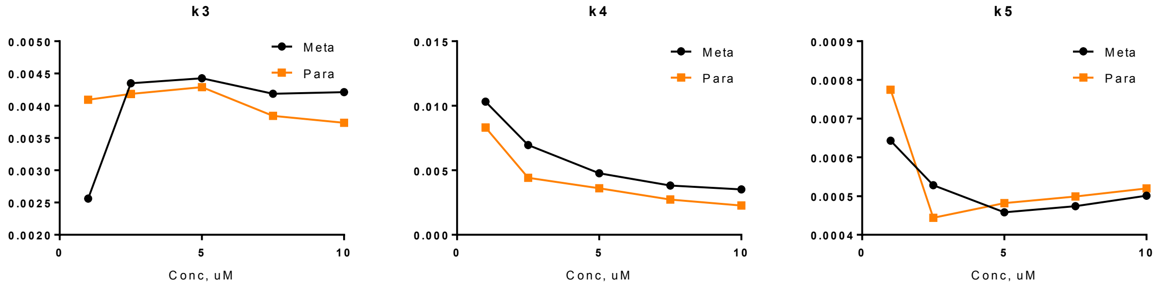

Supplementary Figure 27 | Rate constants not showing a strong concentration threshold. These rate constants show behavior where the parameter mostly trends with concentration and do not show the expected behavior of a differential at low concentration and similarities at high concentration. Parameters k5 and k6 are interesting because they indicate that the losses incurred by the membrane are functions of concentration. These losses may be representations of complex states that are not properly described by a single parameter, which could be cooperative or competing (aggregates, micelle formation, etc).

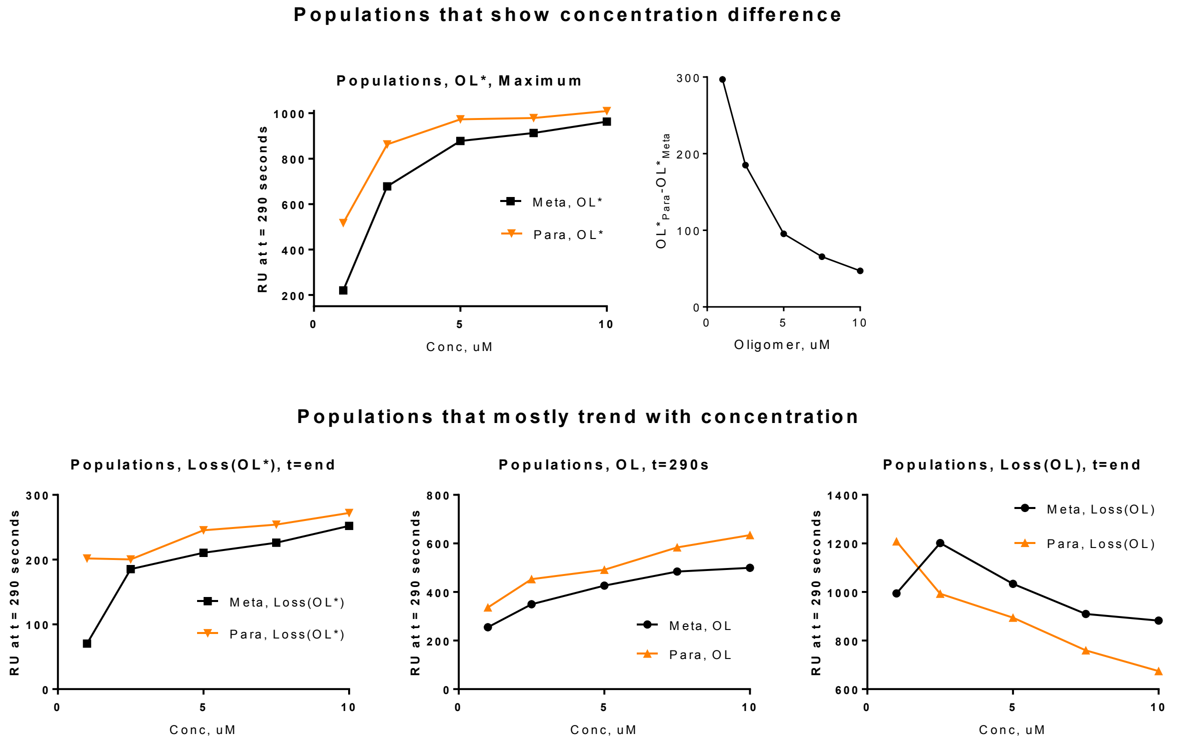

Supplementary Figure 28 | Populations from the Meta and Para model fits versus concentration. (Top) The population of OL* represents the aggregate, and it shows a differential in behavior between the Meta and Para as a function of concentration made more clear when looking at the difference between the two oligomers. **(Bottom)** These populations show behavior where the parameter mostly trends with concentration and do not show the expected behavior of a differential at low concentration and similarities at high concentration. Loss(OL*) does show some indication of trending with OL*, but only around 1μM.

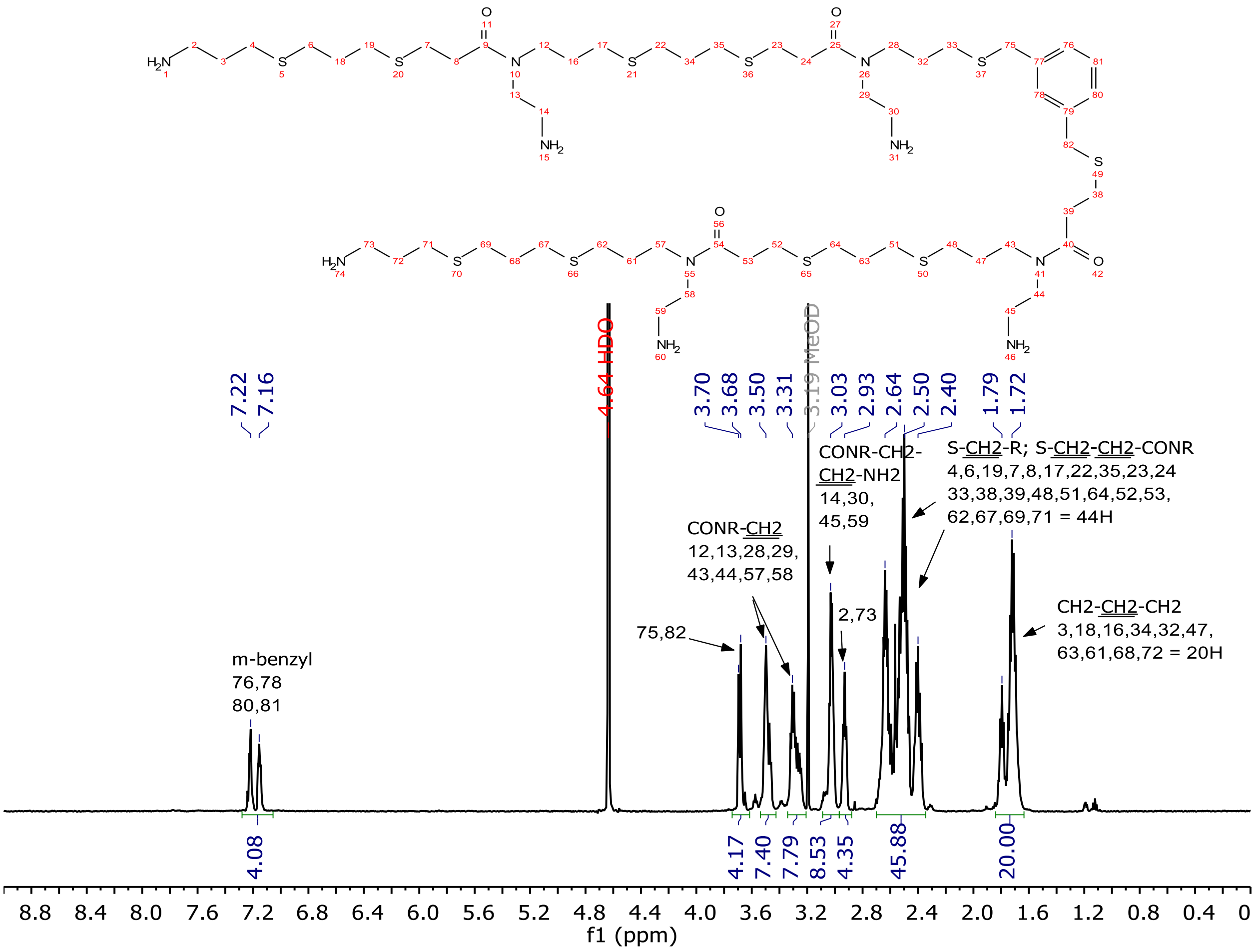

Supplementary Figure 29 | ^{1}H NMR of the Meta in (D_2O, 600 MHz).

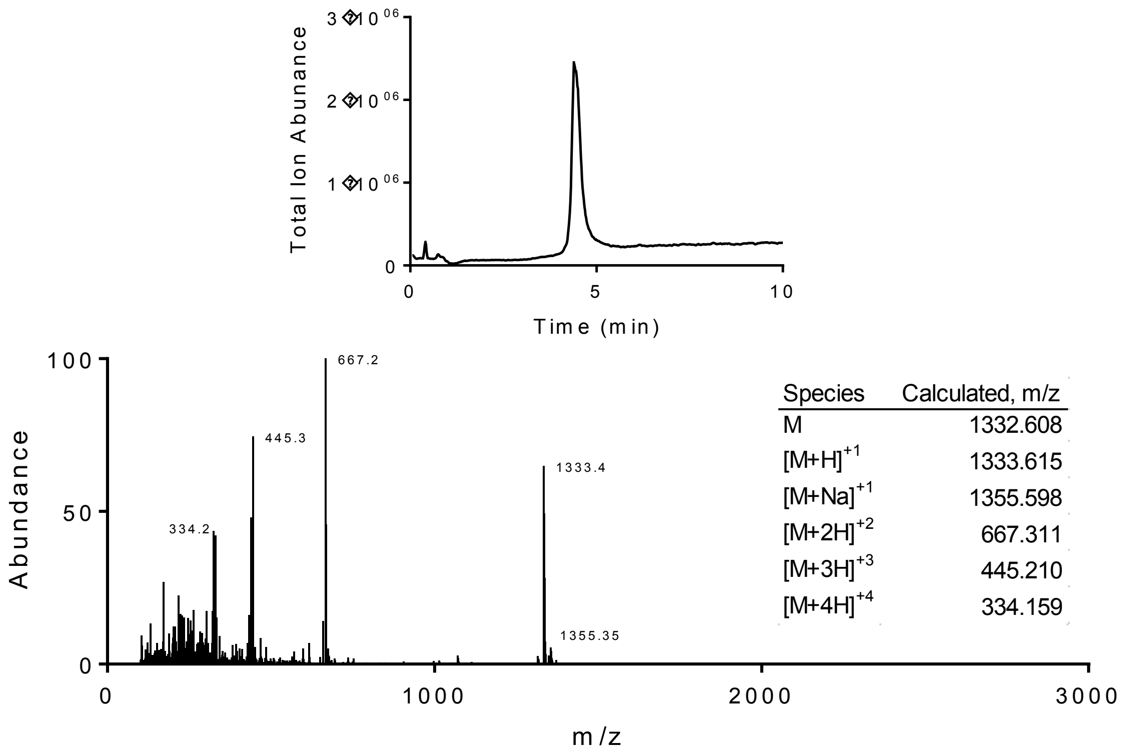

Supplementary Figure 30 | LCMS of the Meta. (Top) Total ion count abundance versus time over a 10 minute gradient. **(Bottom)** Mass spectra confirming the presence of the Meta in the TIC peak.

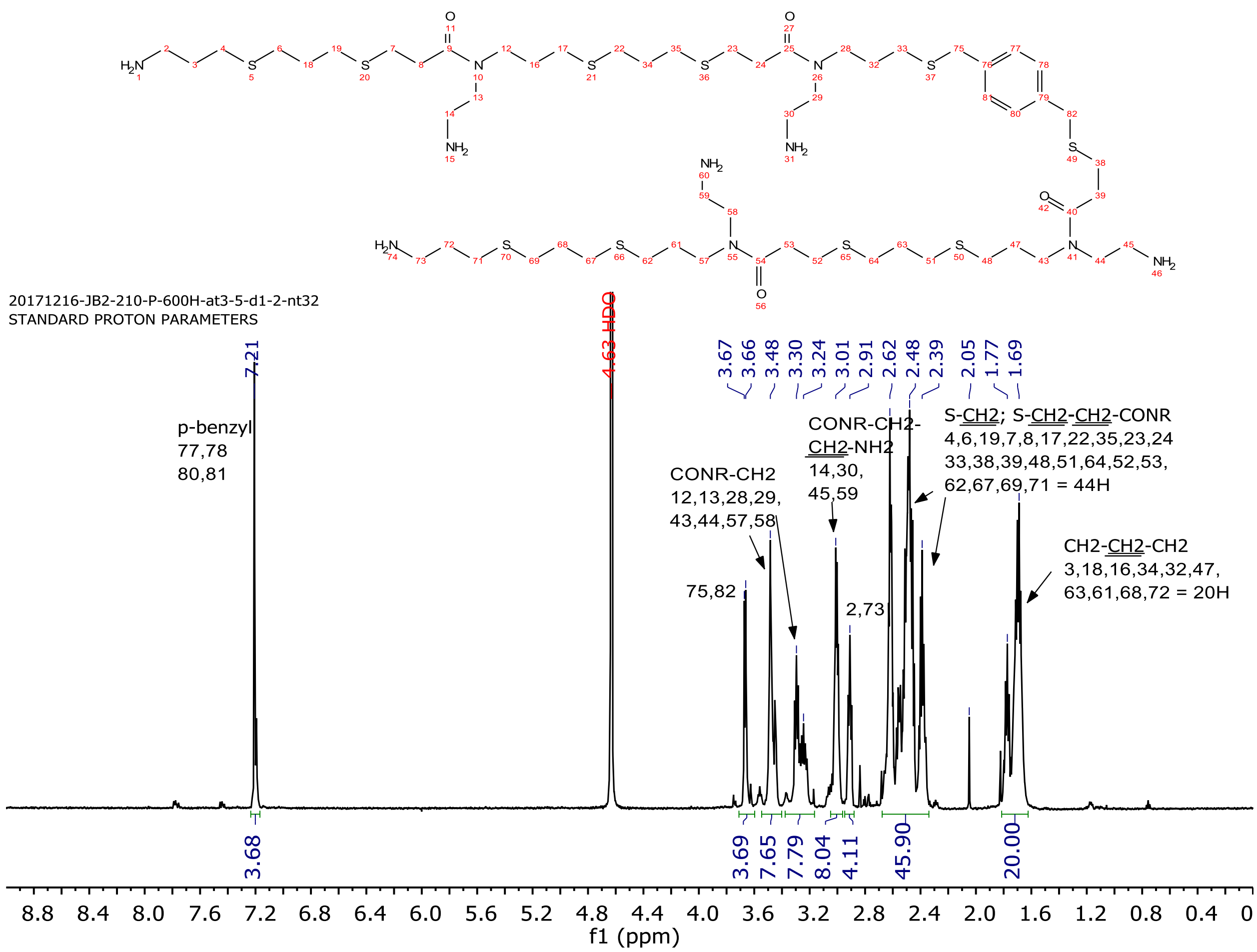

Supplementary Figure 31 | ^{1}H NMR of the Para (D_2O, 600 MHz).

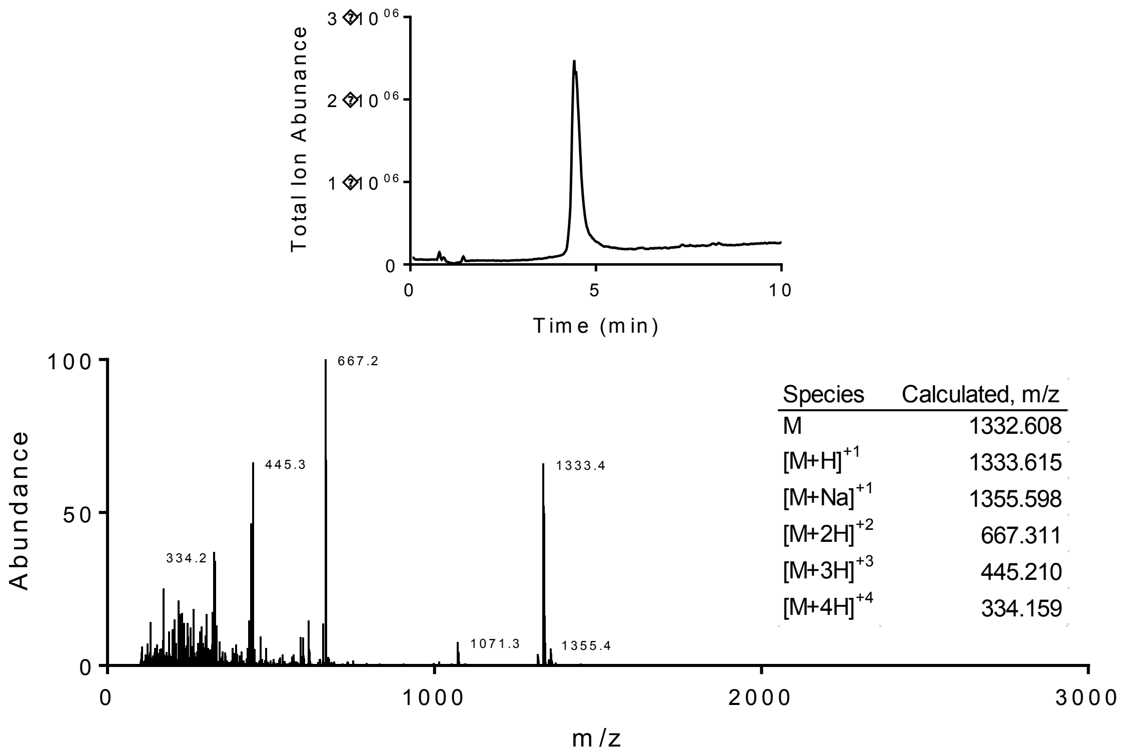

Supplementary Figure 32 | LCMS of the Para. (Top) Total ion count abundance versus time over a 10 minute gradient. **(Bottom)** Mass spectra confirming the presence of the Meta in the TIC peak.

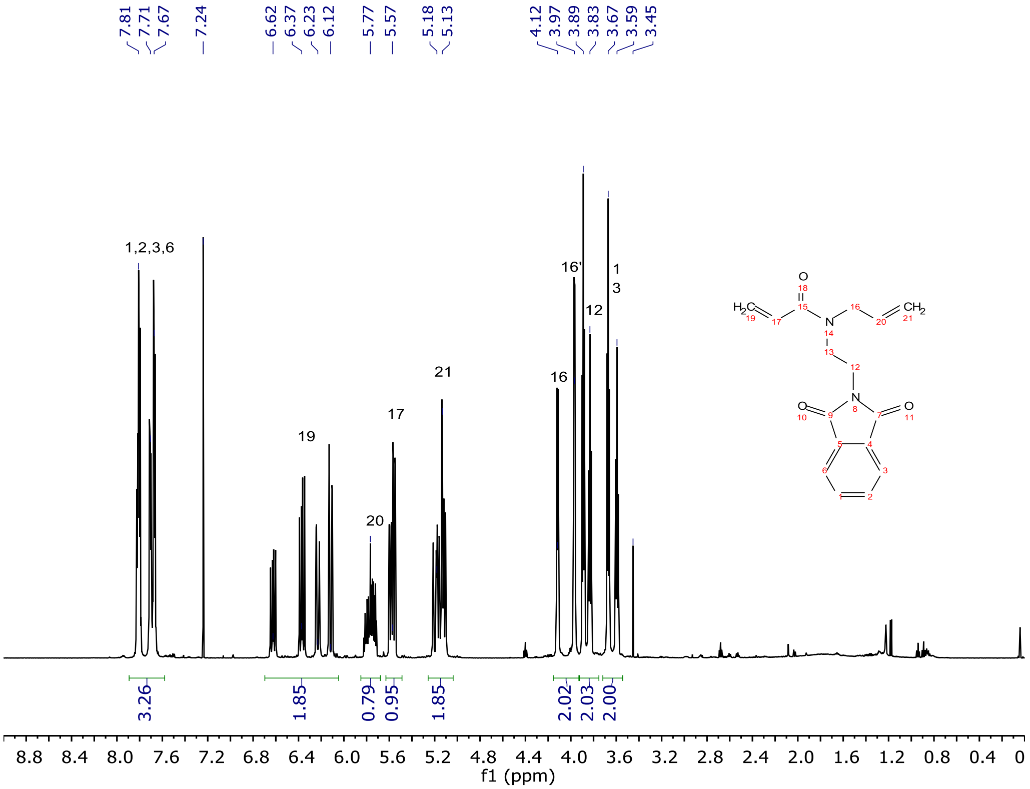

Supplementary Figure 33 | ^{1}H NMR of the (N-phthalimide)ethylene N-allyl-N-acrylamide ($CDCl_3$, 600 MHz)

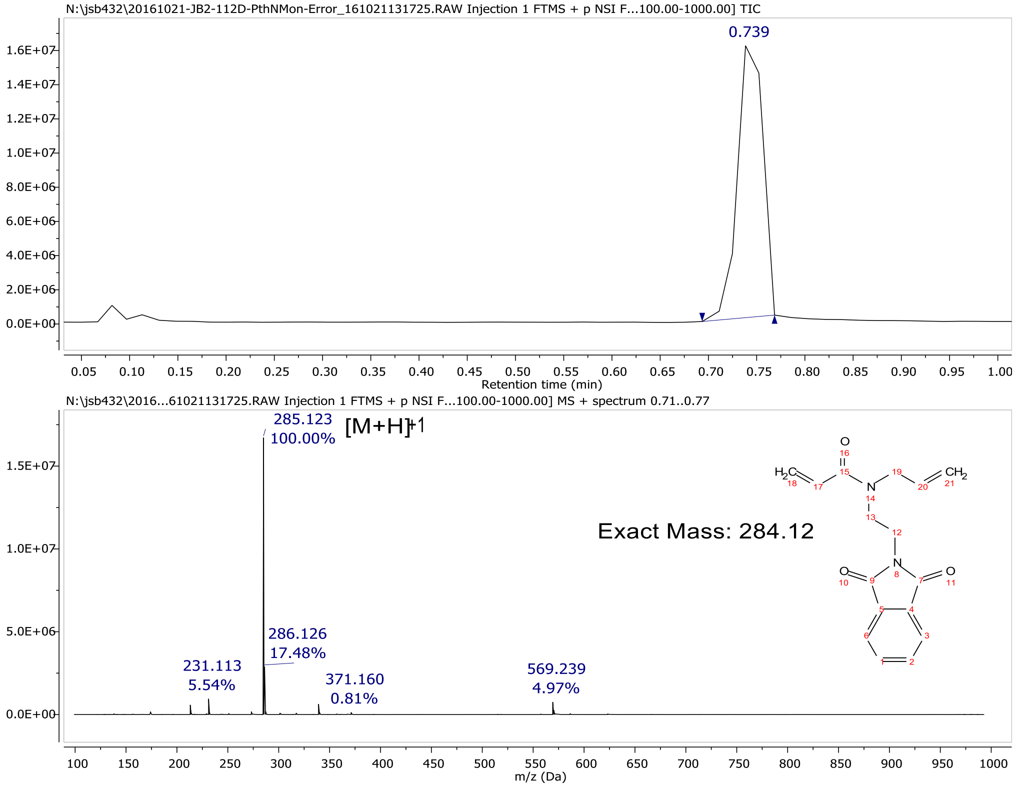

Supplementary Figure 34 | DART HR-MS of the (N-phthalimide)ethylene N-allyl-N-acrylamide

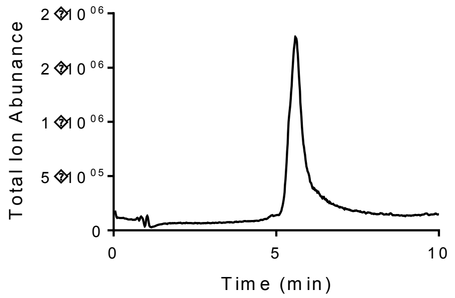

Species	Calculated, m/z	Species	Calculated, m/z	Species	Calculated, m/z
M••	1664.78	M•	1665.79	M	1666.80
[M••+H]$^{+1}$	1665.79	[M•+H]$^{+1}$	1666.80	[M+H]$^{+1}$	1667.80
[M••+Na]$^{+1}$	1687.77	[M•+Na]$^{+1}$	1688.78	[M+Na]$^{+1}$	1689.79
[M••+2H]$^{+2}$	833.40	[M•+2H]$^{+2}$	833.90	[M+2H]$^{+2}$	834.40
[M••+2Na]$^{+2}$	855.38	[M•+2Na]$^{+2}$	855.88	[M+2Na]$^{+2}$	856.39
[M••+3H]$^{+3}$	555.93	[M•+3H]$^{+3}$	556.27	[M+3H]$^{+3}$	556.61
[M••+4H]$^{+4}$	417.20	[M•+4H]$^{+4}$	417.45	[M+4H]$^{+4}$	417.71
[M••+5H]$^{+5}$	333.96	[M•+5H]$^{+5}$	334.16	[M+5H]$^{+5}$	334.37

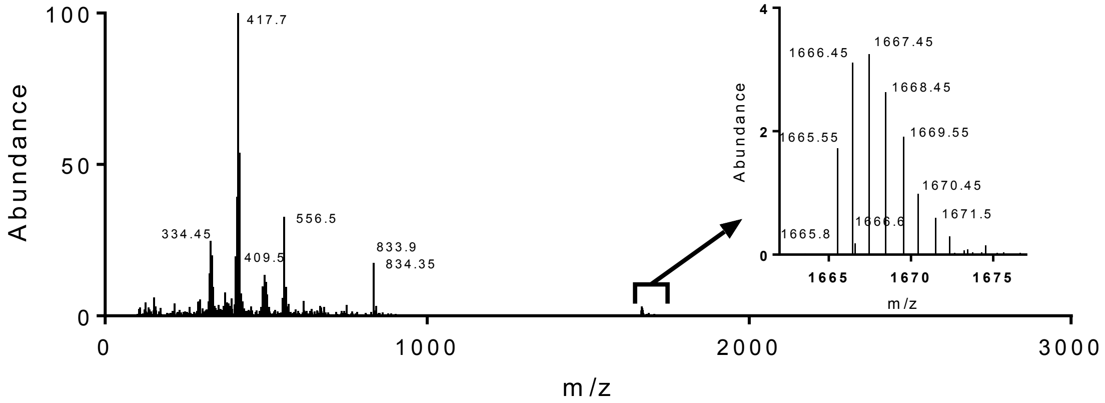

Supplementary Figure 35 | LCMS of the dispin-labeled Meta. (Top, Left) Total ion count abundance versus time over a 10 minute gradient. **(Top, Right)** Expected masses to be observed demonstrating that the spin-label is mostly oxidized (will be reduced before measurement). Masses that were observed experimentally in the mass spectra are highlighted in gray. **(Bottom)** Mass spectra confirming the presence of the Meta in the TIC peak. **(Insert)** Closer examination of the mass spectra shows the parent masses.

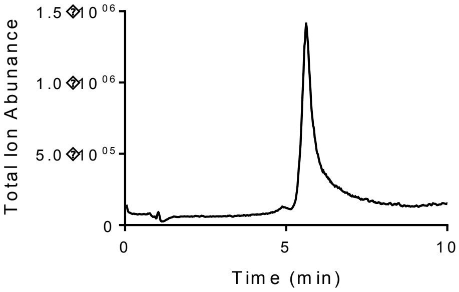

Species	Calculated, m/z	Species	Calculated, m/z	Species	Calculated, m/z
M••	1664.78	M•	1665.79	M	1666.80
$[M••+H]^{+1}$	1665.79	$[M•+H]^{+1}$	1666.80	$[M+H]^{+1}$	1667.80
$[M••+Na]^{+1}$	1687.77	$[M•+Na]^{+1}$	1688.78	$[M+Na]^{+1}$	1689.79
$[M••+2H]^{+2}$	833.40	$[M•+2H]^{+2}$	833.90	$[M+2H]^{+2}$	834.40
$[M••+2Na]^{+2}$	855.38	$[M•+2Na]^{+2}$	855.88	$[M+2Na]^{+2}$	856.39
$[M••+3H]^{+3}$	555.93	$[M•+3H]^{+3}$	556.27	$[M+3H]^{+3}$	556.61
$[M••+4H]^{+4}$	417.20	$[M•+4H]^{+4}$	417.45	$[M+4H]^{+4}$	417.71
$[M••+5H]^{+5}$	333.96	$[M•+5H]^{+5}$	334.16	$[M+5H]^{+5}$	334.37

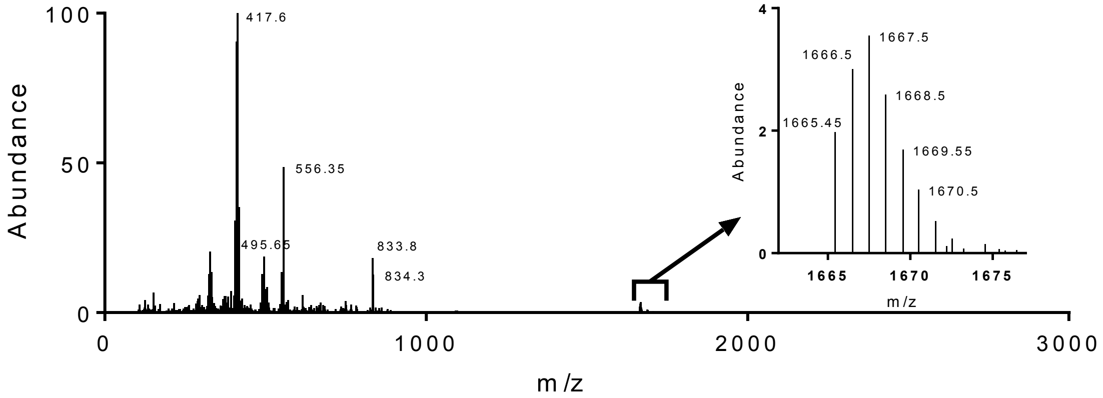

Supplementary Figure 36 | LCMS of the dispin-labeled Para. (Top, Left) Total ion count abundance versus time (10 min gradient). **(Top, Right)** Expected masses; experimentally observed are highlighted in gray. **(Bottom)** Mass spectra confirming the presence of the Meta in the TIC peak, demonstrating that the spin-label is partially oxidized (will be reduced before measurement). **(Insert)** Closer examination of the mass spectra shows the parent masses.

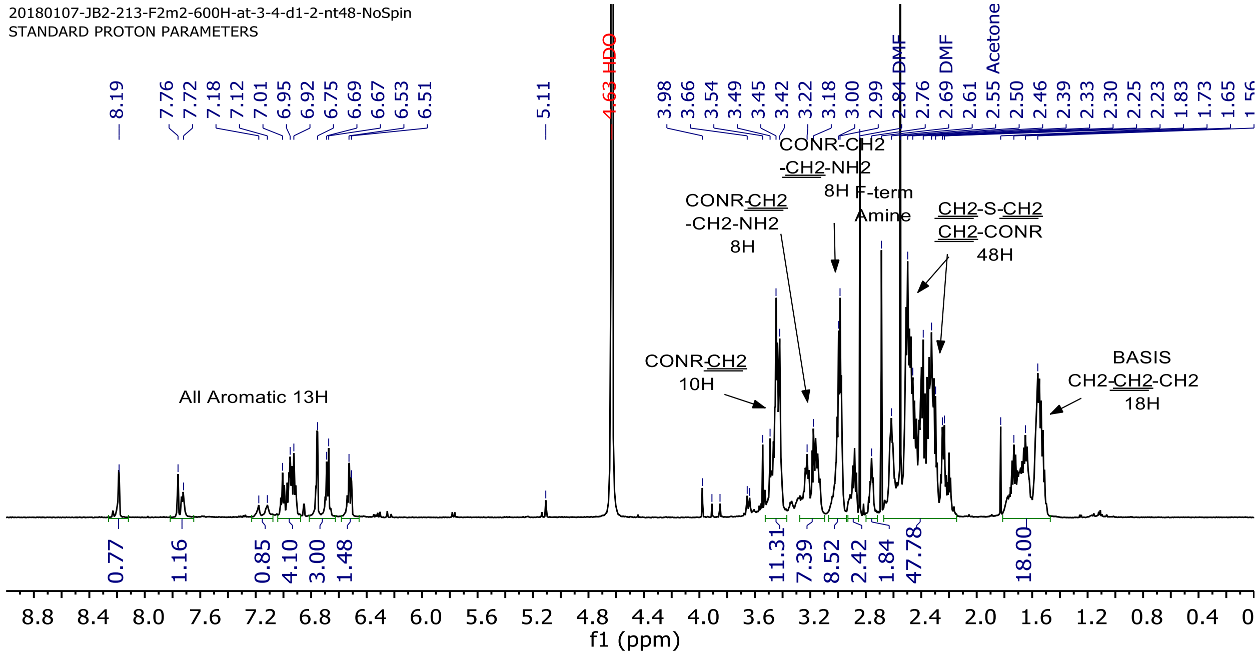

Supplementary Figure 37 | 1H NMR of the fluorescein-labeled Meta (D_2O at 600 MHz).

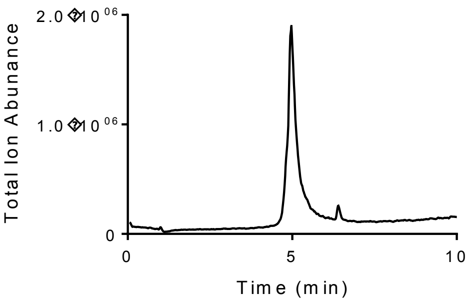

Species	Calculated, m/z
M	1676.64
$[M+H]^{+1}$	1677.647
$[M+Na]^{+1}$	1699.63
$[M+2H]^{+2}$	839.327
$[M+3H]^{+3}$	559.887
$[M+4H]^{+4}$	420.167
$[M+5H]^{+5}$	336.335

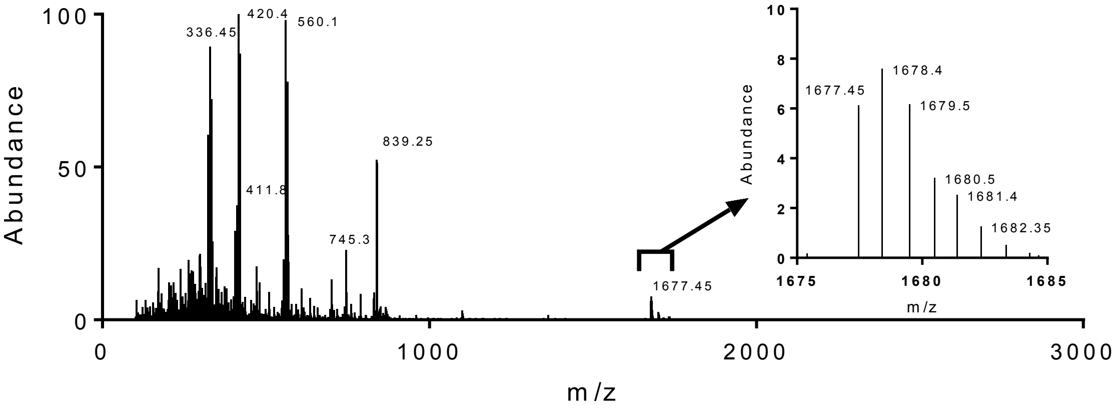

Supplementary Figure 38 | LCMS of the fluorescein-labeled Meta. (Top, Left) Total ion count abundance versus time over a 10 minute gradient. **(Top, Right)** Expected masses to be observed **(Bottom)** Mass spectra confirming the presence of the Meta in the TIC peak. **(Insert)** Closer examination of the mass spectra shows the parent masses.

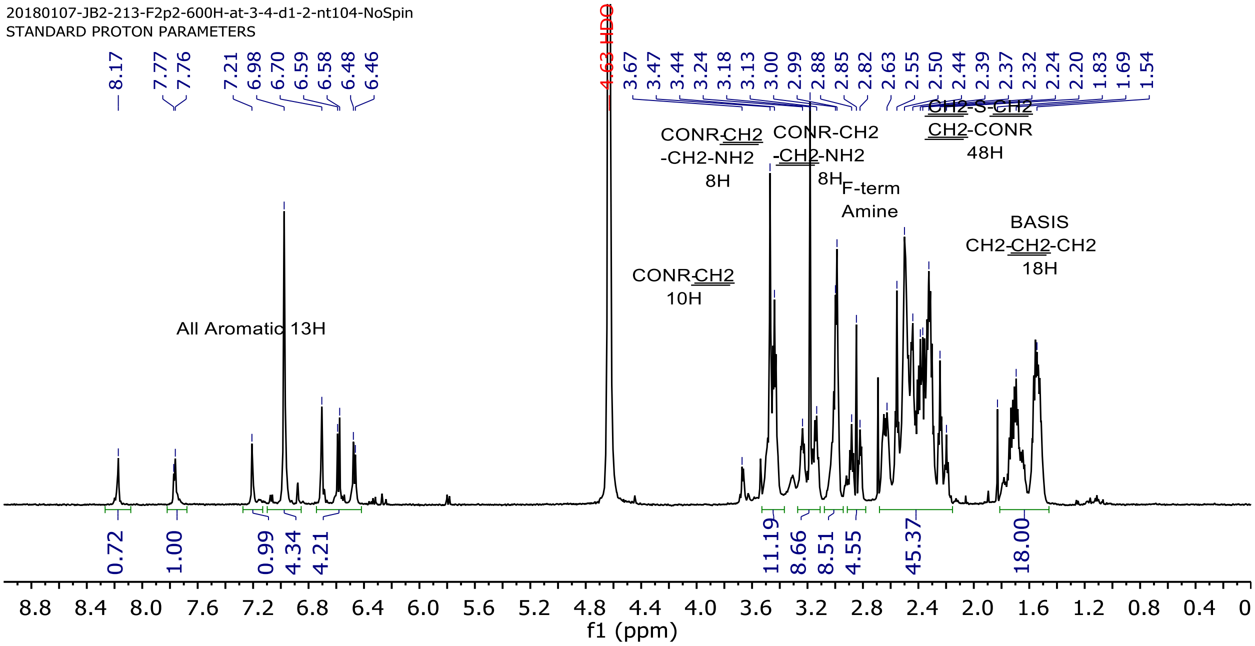

Supplementary Figure 39 | 1H NMR of the fluorescein-labeled Para (D_2O at 600 MHz).

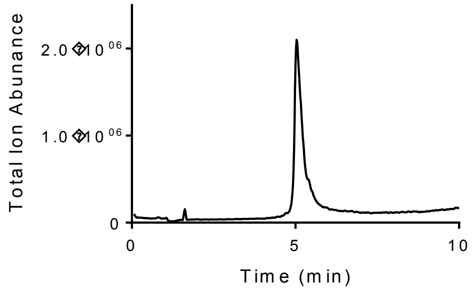

Species	Calculated, m/z
M	1676.64
$[M+H]^{+1}$	1677.647
$[M+Na]^{+1}$	1699.63
$[M+2H]^{+2}$	839.327
$[M+3H]^{+3}$	559.887
$[M+4H]^{+4}$	420.167
$[M+5H]^{+5}$	336.335

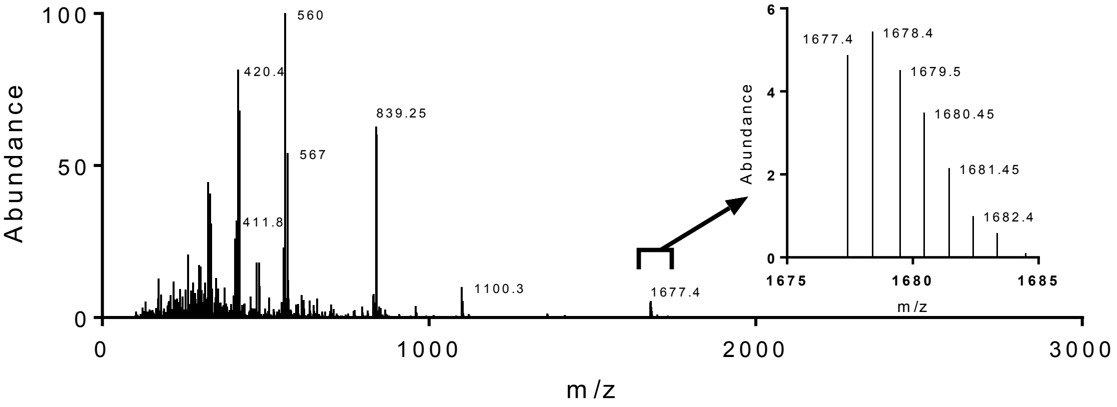

Supplementary Figure 40 | LCMS of the fluorescein-labeled Para. (Top, Left) Total ion count abundance versus time over a 10 minute gradient. **(Top, Right)** Expected masses to be observed **(Bottom)** Mass spectra confirming the presence of the Meta in the TIC peak. **(Insert)** Closer examination of the mass spectra shows the parent masses.

Supplementary Tables.

Supplementary Table 1 | Outputs from Guinier Fits from BioXTAS RAW (1.4.0)

	Concentration, mg/mL	Rg (Å)	Error (Å)	r^2	qRg (low q)	qRg (hi q)	qmin	qmax
**Para**	10	8.568	0.082	0.951	0.454	1.183	0.053	0.138
	5	8.663	0.146	0.843	0.273	1.096	0.032	0.126
	2.5	8.588	0.341	0.523	0.280	1.001	0.033	0.117
	1.25			< 0.3	Not used for Rg analysis due to noise			
**Meta**	10	8.489	0.077	0.952	0.352	1.078	0.041	0.127
	5	8.413	0.121	0.851	0.139	1.087	0.017	0.129
	2.5	8.567	0.196	0.708	0.170	1.102	0.020	0.129
	1.25			< 0.3	Not used for Rg analysis due to noise			

Supplementary Table 2 | All parameters report from model fits of all concentrations with RMSD error

Oligomer	Concentration, μM	k1 (10^4)	k2 (10^{-2})	k3 (10^{-3})	k4 (10^{-3})	k5 (10^{-4})	$C_{Lipid,Total}$	RMSD of Fit
Meta	1	1.00	5.06	2.56	10.3	6.43	3000	9.10
	2.5	1.10	5.28	4.32	6.97	5.25	3000	22.7
	5	0.97	6.84	4.43	4.77	4.58	3000	24.9
	7.5	1.00	9.80	4.19	3.82	4.74	3000	17.3
	10	1.10	13.0	4.21	3.52	5.01	3000	18.2
Para	1	2.65	7.07	4.09	8.31	7.75	3000	11.4
	2.5	2.28	8.00	4.18	4.42	4.44	3000	15.9
	5	1.70	10.0	4.29	3.60	4.82	3000	22.4
	7.5	1.77	14.0	3.84	2.73	4.99	3000	25.2
	10	1.55	15.0	3.74	2.27	5.20	3000	28.6

More details, discussion, methodology, and source code is available in the *Use of MATLAB to model and fit SPR data* section in Supplementary Methods. Parameters from MATLAB fitting shown in table format where RMSD of Fit is defined as $RMSD = \sqrt{\frac{\sum_i^n (Exp_i - Fit_i)^2}{n}}$, where *i* is the index point in time, *n* is the total number of time points, Exp is the experimental data point, and Fit is the fitted model data point at that time index.

Supplementary Discussion.

Compilation of data on lipid composition of *Staphylococcus aureus*.
Several articles were gathered on *Staphylococcus aureus* to understand the lipid composition in growth conditions similar to MIC assay (exponential growth). This includes understanding the amount of neutral lipids present in the membrane, effects of growth phase, and antibiotic resistance mechanism based on the lipid composition. Ultimately, the main goal is to determine a lipid composition to use in membrane mimetic studies. While bacterial membranes are anionic, a simple, fully-charged lipid bilayer would likely be mechanically frustrated from electrostatic repulsion and does not serve as a good mimetic.

Three reports quantified the neutral lipid (NL) component of *S. aureus* to be 19±7% of the membrane. Five other reports mentioned/showed qualitative support of the neutral lipid fraction but did not quantify it. Phosphatidylglycerol (PG) is a major headgroup component in the *S. aureus* membrane with eleven reports quantifying it at an average of 57±5% Cardiolipin (CL) was also quantified in similar reporting to be 16±4% of the membrane. Lastly, lysylphosphatidylglycerol (lysyl-PG or PG) was reported at 16±3% of the *S. aureus* membrane. LPG has an attached lysine via the carboxylic acid, meaning there are two free amines on the structure. The predicted pKa (Marvin Sketch 16.5.23) of these amines is 7.4 (amino group) and 10.21 (functional group), meaning at physiological pH, it has a net +0.5 charge. On a surface, there are additional effects on these pKa numbers and molecular association. LPG has been shown to impart resistance to cationic antimicrobials, including nisin and human defensins in *S. aureus*.[15] However, LPG is expensive (~$200/mg) and its use was prohibitive for study in the scope of this work, which seeks only to understand the interaction of antibacterial oligoTEAs and an *S. aureus* mimetic membrane. LPG within the native *S. aureus* membrane should attenuate the activity of any cationic membrane disrupting agent, but likely does not broadly change the mechanics of interaction.

Two lipid compositions were thus utilized:

1) 70% PG and 30% CL or 7:3 PG:CL
   Reflects the often larger amount of PG to CL in the *S. aureus* while still being significantly different than 100% PG
2) 20% neutral lipid (e.g., diacylglycerol), 55% PG, and 25% CL or 4:5:11 NL:CL:PG

List of publications that detail the lipid composition in S. aureus:

Publication:	Lipid compositions known in MRSA:
P Beining et al (1975)[16]	(table below)
J Cheng et al (2011)[17]	Says that 1:1 (TO)CL:POPG and 1:1 POPC:POPG are acceptable model membranes (Gram positive)<br>1:1 POPE:POPG is not though
N Mishra and A Bayer (2013)[18]	Carotenoids (neutral) present, but unquantified<br>LPG: 13.9%; PG: 79.6% CL: 6.5%<br>Measured across 9 strains.

P Beining et al (1975)[16]:

	Value	Percent
MonoGlu	0.003	0.3%
DiGlu	0.026	2.6%
PhosGlu	0.004	0.4%
PG	0.306	30.1%
Cardiolipin	0.224	22.1%
Carotenoid	0.132	13.0%
Vitamin K	0.320	31.5%
Sum	1.015	100%

C Haest et al (1972)[19]	Neutral lipids mentioned, but not quantified<br>Carotenoids/VitK not mentioned<br>LPG: 38% PG: 57% CL: 5%
Y Kanemasa et al (1972)[20]	CL increases and PG decreases as the *S. aureus* transitions from exp to stationary phase.<br>Neutral lipids mentioned/shown (TLC ~50%)<br>Mid-log, CL: 11% PG 72% LPG 11% Other 6%
E Garcia-Fernandez et al (2017)[21]	Emphasizes importance of carotenoids in beta-lactam (methicillin) resistance in *S. aureus*<br>No quantification of lipid compositions.
M Tsai et al (2011)[22]	Phospholipids only:PG: 43% CL: 46% LPG: 11%
N Mishra et al (2011)[23]	Carotenoids are in higher proportion in MRSA compared to regular *S. aureus*.<br>Decreases membrane fluidity (more stiff, like CL)<br>Thought to be mechanism for resistance for AMPs
M Hayami et al (1979)[24]	Neutral lipids mentioned 5-10% of total<br>Phospholipids reported:<br>CL: 20% PG: 43% LPG: 32% Other 5%
D White and F Frerman (1967)[25]	Neutral lipids (VitK, Glycolipid) shown
D White and F Frerman (1968)[26]	Neutral lipids are 5-10% of lipids<br>Experimentally observed 12.5%<br>LPG: 9% PG: 60% CL: 19%<br>The amount of neutral lipids depends on the growth media (especially if serum is present)
S Short and D White (1970)[27]	During exp phase PE: 2%<br>and stationary phase PE: 14%

Supplementary Methods.

Most all tabulated data was processed with GraphPad Prism 7.01. (1x) PBS was pH 6.8 unless otherwise stated. NMR spectra were recorded on an INOVA 600 MHz spectrometer and analyzed by MestReNova (version 10.0.0). ^{1}H NMR chemical shifts are reported in units of ppm relative to the deuterated solvent.

LCMS experiments were carried out on an Agilent 1100 LCMS system with a Poreshell 120 EC-C18 (3.0x100mm, 2.7um) column monitoring at 210nm in positive mode for detection. Solvents for LCMS were water with 0.1% acetic acid (solvent A) and acetonitrile with 0.1% acetic acid (solvent B). A flow rate of 0.6 mL/min was used with a gradient starting at 5% solvent B, followed by a linear gradient of 5% to 100% solvent B over 10 min, 100% solvent B for 2 min, before returning to 5% solvent B until equilibrated (3 min).

HPLC purification was performed on an 1100 Series Agilent HPLC system using a reverse phase Agilent Eclipse XDB-C18 column (9.4x250mm, 5µm) at 40°C and collected using an automated fraction collector. Solvents for HPLC were water with 0.1% trifluoroacetic acid (solvent A) and acetonitrile with 0.1% trifluoroacetic acid (solvent B). Compounds were eluted with either a 3.17 %B/min gradient or a 1.25 %B/min gradient at 4mL/min.

Pure and chemically confirmed oligoTEAs were quantified by being lyophilized into a tared microcentrifuge tube. Single stock solutions were prepared at either 1 or 5 mM in ultrapure water and used for all experiments.

Statistical analysis was performed on specific data sets as described in the main text. Means are defined as $\bar{x} = \frac{1}{n}\sum_i x_i$; standard deviation defined as $\sigma = \sqrt{\frac{\sum_i (x_i - \bar{x})^2}{n}}$; and pooled variance was defined as $s^2 = \frac{\sum_i (n_i - 1)\sigma_i^2}{\sum_i (n_i - 1)}$. Where used, confidence intervals are provided as a measure of uncertainty.

Additional methods for oligothioetheramide assembly.

General method for fluorous allyl amine synthesis:

2-[2-(1H,1H,2H,2H-Perfluoro-9-methyldecyl)isopropoxycarbonyloxyimino]-2phenylacetonitrile (fluorous tag) was dissolved in THF (15mg/mL). Two equivalents of allyl amine and two equivalents of triethylamine were added and stirred at RT for at least 3 hours. The THF was completely removed and the reaction mixture was fluorous purified to yield fluorous allyl amine as a light yellow solid.

General method for thiolene reaction:

Two equivalents of 1,3-propanedithiol or meta- or para-benzyl dimethanethiol and 2,2-dimethyoxy-2-phenylacetophenone (DMPA 10 mol% of dithiol) were added to a solution of fluorous olefin in methanol or 1:1 acetone:methanol and irradiated at 20mW/cm^2 for 270s. The fluorous thiol product was purified by FSPE.

General method for thiol-Michael addition:

Two equivalents of the Boc-amine ethylene N-allyl-N-acrylamide was activated by dimethylphenyl phosphine (DMPP, 5mol% of monomer) and added to the fluorous thiol (50-300mM in methanol or 1:1 methanol:acetone). The reaction completion was tracked by thiol detection via an assay with 2,2' dithiodipyridine (DTDP) as described.[1]

Hemolysis Assay.

200 µL of red blood cells (Innovative Research Novi, MI) were washed twice with 500 µL PBS by centrifugation (5 min at 500g) and resuspended in PBS at 4 v/v%. OligoTEA solutions or controls were mixed 1:1 with the RBC solution in a v-bottom 96-well plate (100 µL total). The resulting mixture was incubated at 37°C for 1 h and then centrifuged (Beckman Coulter, 5 min at 1000g) at 4 °C. A total of 75 µL of supernatant was transferred to a new plate. Hemolysis was measured via absorbance of released hemoglobin at 540 nm on a TECAN Infinite M1000 PRO Microplate reader (Männdorf, Switzerland) and normalized to 0.1% Triton-X (100%) or PBS buffer (0%). All experiments were performed in duplicate.

Spin-labeling the oligomer scaffold.

With the use of several primary amine groups in this scaffold, an amine protecting strategy orthogonal to base (synthesis of monomer), nucleophile (phosphine of thiol-Michael), UV of thiolene, and acid (TFA, fluorous tag deprotection) had to be utilized to enable spin labeling. The phthalimide-protected amine N-allyl-N-acrylamide was thus developed (Figure S3). Generally, monomers are produced by alkylation (allyl group) and then acylation (acrylamide) or vice versa starting with either functional amine or alkyl halide. However, insufficient yield was obtained from the alkylation and then acylation of a phthalimide protected ethylene diamine. Also, the phthalimide was unstable in the presence of NaH, needed to for the acylation-then-

alkylation route. Deprotection of the N-Boc ethylene N-allyl-N-acrylamide with TFA and re-protection with phthalic anhydride was not feasible due to immediate polymerization.

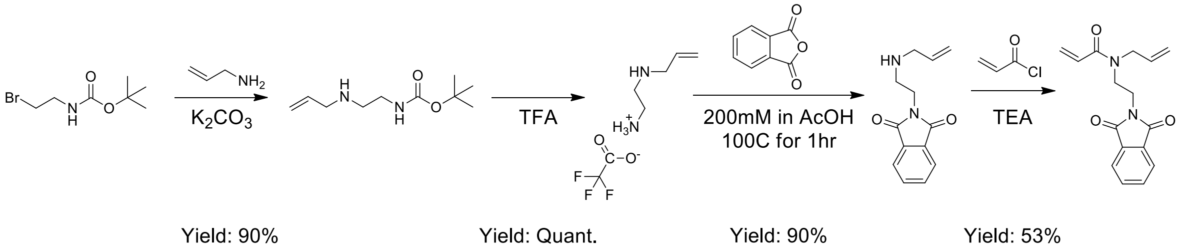

Thus, the following scheme was developed (Figure S3, shown again above): 1) alkylation of N-Boc ethylene bromide with 50 eqv of allyl amine 5eqv of K_2CO_3 purified by filtration, 2) deprotection with 200 mM 1:1 TFA:DCM stirred at RT for 1 hour, 3) treatment with 1eqv phthalic anhydride 200 mM in acetic acid at 100C for 1 hour, dried, precipitated in water and dried, and 4) acylation as previously described[2] and flash purified, eluting at 60% ethyl acetate in hexanes. In Step 3, the selectivity of the phthalic anhydride to protect primary amines over secondary amines was critical, but was reported in literature before.[3,4]

With the N-phthalimide ethylene N-allyl-N-acrylamide, assembly of the antibacterial oligoTEA was completed as previously described.[5] The standard TFA deprotection then yields two primary amines for conjugation with a Proxyl-NHS (Toronto Research Chemicals, North York, Canada) with conditions previously reported.[1] Full deprotection of the oligomer (4 phthalimide groups) was modest with hydrazine, even with heat. Thus, a previously reported route of reduction with sodium borohydride and hydrolysis with acid[6] was optimized to utilize a stronger acid and less heat (Figure S4, shown again below). The oligomer was purified by HPLC and verified by LCMS (Figure S34,S35). The paramagnetic label prevents against full ^{1}H NMR of the compound.

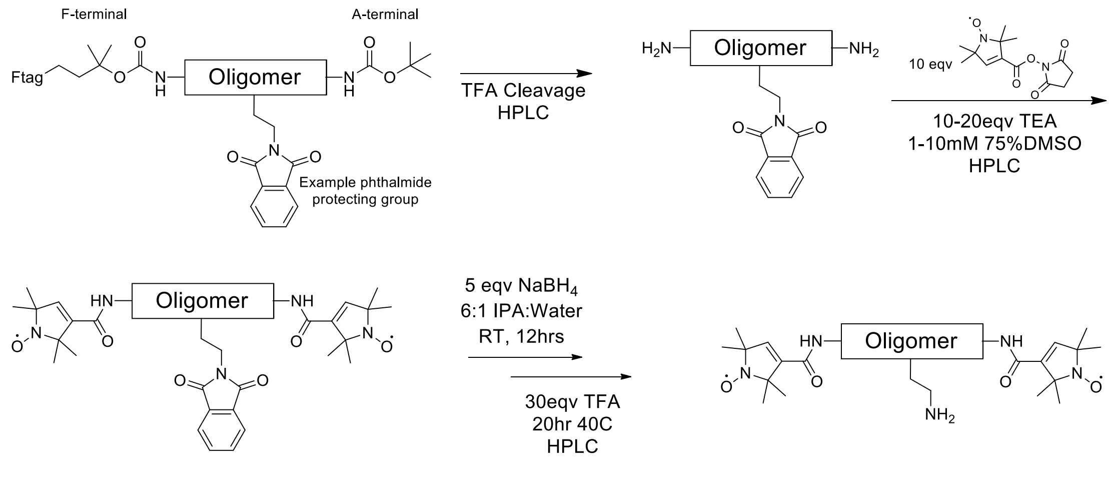

Use of VT-PFG NMR, DEER EPR, and Stokes-Einstein-Sutherland Equation.

This method to measure the size and aspect ratios of flexible macromolecules has previously been discussed extensively[1] and makes use of work from Stokes-Einstein-Sutherland (SES), Chen and Chen[7], and G. de la Torre[8], and F. Perrin[9–11] with a review by A. Macchioni[12].

$$D_t = \left(\frac{k_B}{c(r_H)f_S(p)\pi r_H}\right)\frac{T}{\eta}$$

$$slope \equiv \left(\frac{k_B}{c(r_H)f_S(p)\pi r_H}\right) \rightarrow \frac{k_B}{(slope)(\pi)} = c(r_H)f_S(p)r_H$$

The SES equation above relates the Boltzmann temperature and the translational molecular diffusion where k_B is the Boltzmann constant, T is the temperature in Kelvin, r_H is the prospective hydrodynamic radius, η is the dynamic viscosity, c is a size-dependent modification to transition between the slip/no-slip boundary conditions,[7] p is the geometrically defined aspect ratio, and f_s is the shape-modified friction factor.[9–11] By measuring the diffusion by variable temperature PFG-NMR, the two remaining unknowns in this equation of hydrodynamic radius and shape are constrained. This is especially sensitive with the slope of the normalized temperature (T/η) is used. Then, the end-to-end distance can serve as a prospective diameter and allow the equation to be solvable, revealing the hydrodynamic radius and aspect ratio given a geometric shape, either an ellipsoid[9–11] or rod.[8] For this analysis, a prolate ellipsoid was assumed and the end-to-end distance was assumed to describe the longer dimension of the ellipsoid. The final equation was solved using fsolve in MATLAB R2017a with MaxIter = 800, MaxFunEvals = 200, a tolerance of 10^{-25}, and a multiplier on the residual of 10^{15} as it solved for meters.

Small unilamellar vesicles (SUVs) preparation.
1-2-dioleoyl-sn-glycerol (18:1 DG), 1-palmitoyl-2-oleoyl-sn-glycero-3-phosphocholine (16:0-18:1 PC, POPC), 1-palmitoyl-2-oleoyl-sn-glycero-3-phosphoglycerol (16:0-18:1 PG, POPG), 1',3'-bis[1,2-dipalmitoyl-sn-glycero-3-phospho]-sn-glycerol (16:0 cardiolipin, TPCL), 1',3'-bis[1,2-dioleoyl-sn-glycero-3-phospho]-sn-glycerol (18:1 cardiolipin, TOCL) were purchased from Avanti Polar Lipids, Inc. Cholesterol (≥99%) was purchased from Sigma Aldrich. SUVs were prepared similar to the Avanti Polar Lipids protocol. Lipids were combined from chloroform stock solutions and dried using dry nitrogen at room temperature and dried for at least 6 hours at ≤10 mtorr. The dried lipids were suspended in 1x phosphate buffered saline (PBS) by vigorous vortexing for 5 minutes. Light sonication was needed for any lipid mixture with TPCL. The suspension was gently mixed overnight to age. The suspension was then sonicated for 10-30 minutes in a water bath (RT) using a QSonica Q125 probe-tip sonicator (20kHz) at 50% power, pulsing for 7 seconds with a 2 second rest. The resulting translucent suspension was then centrifuged at 17k RCF for 7 minutes to remove any metal particles. The solution was then filtered through a 0.2 μm PES filter. Dynamic light scattering (DLS) was then completed on a Malvern Zetasizer XS determining the hydrodynamic size to be 20-80 nm (Figure S8a). Particle concentrations such that the attenuator was automatically set at 7 or 8, preset parameters of a liposome (RI 1.34) dispersed in PBS (0.9103 cP at 25C) were used. Zeta potential measurement was completed at the same particle concentration (Figure S7b). With the exception of TPCL lipid mixes (1 week), SUVs were stored at 4C and seen to be stable for approximately 3-4 weeks.

Particle analysis in ImageJ to quantify number and size of aggregates.

Thresholding was performed to select the brightest pixel populations using the “Triangle” method.[29] While ImageJ was set to analyze particles from 0.0-10 μm, the smallest aggregates of ~0.1 μm were at the resolution and threshold limit, limiting the accuracy of their quantification in number and size by generally undercounting. Aggregates 0.2 μm and greater were reliably counted. The upper limit of particle analysis was 10 μm, with most oligomer induced aggregates ranging from 0.2-7.5 μm for the Meta and 0.2- 5μm for the Para. For equilibrium and kinetic counts, the total count of particles was subtracted from the number initial defects (e.g. spots) present on the membrane to determine the number of aggregates that formed during oligomer exposure.

Surface plasmon resonance (SPR).
SPR was completed using a Biacore 3000 with an L1 Chip at 25C modified slightly from manufacturer protocol. Before use, desorb, sanitize, and an overnight wash with ultrapure water were completed. 1x pH 6.8 PBS was used throughout in all runs and solutions. Conditioning of the L1 chip was completed at the beginning and end of each run with 7uL 40mM octyl-β-glucopyranoside at 10uL/min (Alfa Aesar). Additional manufacturer recommended washes were used. SUV capture was performed at 5 uL/min for 10 minutes to ensure surface saturation. Control runs of 1 minute pulses of 10mM NaOH showed little change in the baseline and ensured minimal formation of multilayer membranes. At an equilibrated flow of 30 uL/min, samples were injected (kinject) for 5 minutes and dissociated for 6 minutes with PBS.

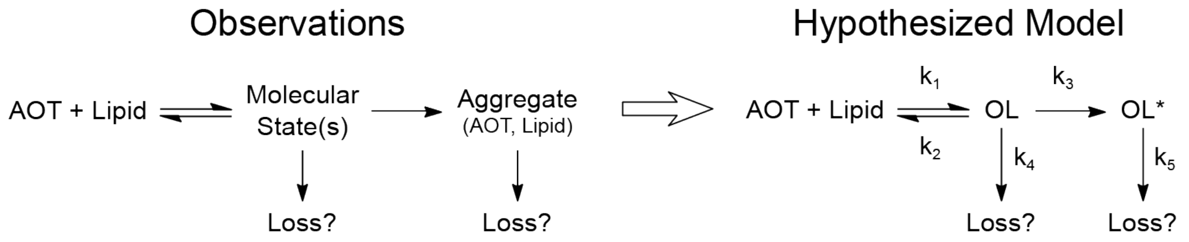

$$\frac{dOL}{dt} = k_1[AOT][C_{Lipid,Total} - OL - OL^* - Loss(OL) - Loss(OL^*)] - k_2 OL - k_3 OL - k_4 OL \quad (1)$$
$$\frac{dOL^*}{dt} = k_3 OL - k_5 OL^* \quad (2)$$
$$\frac{dLoss(OL)}{dt} = k_4 OL \quad (3)$$
$$\frac{dLoss(OL^*)}{dt} = k_5 OL^* \quad (4)$$
$$\frac{dSignal_{SPR}}{dt} = \frac{dOL}{dt} + \frac{dOL^*}{dt} \quad (5)$$

Equations S1 | Ordinary differential equations from kinetic framework. (top) Excerpt of Figure 5a of translated to mass action kinetics rates (Equations 1-5). SPR observes effective refractive index changes near the gold sensor surface, encoding both mass and structural changes. Thus, the SPR cannot observe material lost from the surface seen in Equation 3 and 4 and Equation 5 sums up the combined observations.

Use of MATLAB to model and fit SPR data.
Fitting of the kinetic rates to the SPR sensorgram data was completed in MATLAB R2017a (9.2.0.538062) using lsqcurvefit. The function was two separate nested ODEs, one each for the association and dissociation phases where $C_{oligoTEA}$ was the designated concentration and 0uM,

respectively. The ODEs were numerically solved by ode15s with RelTol and AbsTol as 1e-8. Convergence of lsqcurvefit was seen typically in less than 1k function iterations to a resnorm less than 1e6 to capture the behavior of the curve. Kinetic rate parameters k_1,k_2,k_3,k_4, and k_5 were not bounded, while $C_{Lipid,total}$ was kept constant (3000 ± <0.01%). $C_{Lipid,total}$ should be dependent on the physical number of binding spots of the lipid surface, only changing based on SUV lipid composition, not the oligomer concentration (only the *S. aureus* lipid composition was fit). $C_{Lipid,total}$ must be greater than the highest RU value in the experimental data. For example, $C_{Lipid,total}$ must be at least ~1650 RU worth of lipid in order to fit the 10μM Para from Figure S19. $C_{Lipid,total}$ scales the curve response, with little change to the curve shape. Since the kinetic rates significantly affect curve shape, $C_{Lipid,total}$ was held constant to allow comparison of kinetic rates.

Supplementary Software.

MATLAB Code.

The code is broken into a script that calls a custom function. Within the function are the ODEs for association and dissociation ([Oligomer] = 0) are called by the script to be numerically integrated in a sequential manner.

Script:

```
close all
clear all

global M1 M2 M3 M4 MSPR xswitch

% point where buffer flows in
xswitch = 296;

filename = 'Para_2p5uM_SAureus.txt';
fileopen=fopen(filename,'r');
filein = fscanf(fileopen,'%f',[2 Inf]);
data = filein';
fclose('all');
t = data(:,1);
y = data(:,2);

%initial values of k
%k0=[2e3; 0.01; 0.01; 0.01; 0.005; 1.5e+4]; Working with Melittin
%   k1     k2      k3      k4       k5      k6       LipTot
k0=[2.3e4; 0.078; 0.0041;  0.0;  0.0045;  0.00042;  3e3];

% %bounds for parameters
%        k1      k2    k3     k4      k5     k6       LipTot
lb = [1e2;  1e-4;  1e-8;  0;      0;     0;        2.95e3]; %lower boundary for
parameters
ub = [1e5;    1;   1;      1e-12;  0.1;   0.01;   3.05e3]; %upper boundary for
parameters

%Plot curve with initial values
timestmp = datestr(now);
timestmpfix = regexprep(timestmp,'[:]','');
figure(1), clf
set(gcf,'units','points','position',[100,100,800,600])
f1 = plot(t,y,'o',t,Funct2StateWithLossOn1And2(k0,t),t,M1,t,M2,t,M3,t,M4);
xlabel('Time, s')
ylabel('RU')
```

```
hold off
set([f1(2)],'LineWidth',2)
set([f1(3)],'LineWidth',2)
set([f1(4)],'LineWidth',2)
set([f1(5)],'LineWidth',2)
set([f1(6)],'LineWidth',2)
legend('Exp Data','Fit','Component 1, M1','Component 2,
M2','Loss1','Loss2','location','best')
str = {['k(1) ', num2str(k0(1))];...
         [' k(2) ',num2str(k0(2))];...
         [' k(3) ',num2str(k0(3))];...
         [' k(4) ',num2str(k0(4))];...
         [' k(5) ',num2str(k0(5))];...
         [' k(6) ',num2str(k0(6))];...
         [' k(7) ',num2str(k0(7))];...
         mfilename};
dim = [0.2 0.06 0.3 0.45];
annotation('textbox',dim,'String',str,'FitBoxToText','on')
%ylim([0 2000])
saveas(figure(1),[filename(1:end-4),' ',timestmpfix],'png')

%options for running algorithms
options = optimoptions('lsqcurvefit','Algorithm','trust-region-reflective');
options.OptimalityTolerance = 1.0000e-09;
options.FiniteDifferenceStepSize = 1; % default is eps
options.FiniteDifferenceType = 'central';
options.Diagnostics = 'on';
options.Display = 'iter';
options.FunctionTolerance = 1.0000e-016;
options.FunValCheck = 'on';
options.MaxFunEvals = 5000;
options.MaxIter = 1000;
options.StepTolerance = 1.0000e-012;

% % fit the data
[k,resnorm,output,exitflag] =
lsqcurvefit(@Funct2StateWithLossOn1And2,k0,t,y,lb,ub,options);
figure(2), clf
set(gcf,'units','points','position',[100,100,800,600])
f2 = plot(t,y,'o',t,Funct2StateWithLossOn1And2(k,t),t,M1,t,M2,t,M3,t,M4);
xlabel('Time, s')
ylabel('RU')
hold off
set([f2(2)],'LineWidth',2)
set([f2(3)],'LineWidth',2)
set([f2(4)],'LineWidth',2)
set([f2(5)],'LineWidth',2)
set([f2(6)],'LineWidth',2)
legend('Exp Data','Fit','Component 1, M1','Component 2,
M2','Loss1','Loss2','location','best')
loc = [.65 .1 .9 .4];
str = {['k(1) ', num2str(k(1))];...
         [' k(2) ',num2str(k(2))];...
         [' k(3) ',num2str(k(3))];...
         [' k(4) ',num2str(k(4))];...
         [' k(5) ',num2str(k(5))];...
         [' k(6) ',num2str(k(6))];...
```

```
        [' k(7) ',num2str(k(7))];...
        [' Residual ',num2str(resnorm)];...
        mfilename};
annotation('textbox',loc,'String',str,'FitBoxToText','on')
saveas(figure(2),[filename(1:end-4),'_Output',timestmpfix],'png')

newdata(:,1) = t;
newdata(:,2) = Funct2StateWithLossOn1And2(k,t);
newdata(:,3) = M1;
newdata(:,4) = M2;
newdata(:,5) = M3;
newdata(:,6) = M4;
endfilename = [filename(1:end-4),timestmpfix,'Output.txt'];
endfile = fopen(endfilename,'w');
dlmwrite(endfilename,newdata,'delimiter','\t')
fclose('all');
```

Function:

```
function R = Funct2StateWithLossOn1And2(k,t)

global M1 M2 M3 M4 MSPR xswitch

Cp=2.5*10^-6;    %effective molar concentration of peptide in solution
ode_options = odeset('RelTol', 1e-8, 'AbsTol', 1e-08); %, 'Stats','on');
y0 = [0;0;0;0;0]; %initial conditions; set by number of ODEs used (including
dSPR/dt)
[~,Fit1]=ode15s(@DiffEq1,t(1:xswitch),y0,ode_options);

yswitch = [Fit1(end,1),Fit1(end,2),Fit1(end,3),Fit1(end,4),Fit1(end,5)]; %Cp
should now be zero
[~,Fit2]=ode15s(@DiffEq2,t(xswitch+1:end),yswitch,ode_options);

Fit = cat(1,Fit1,Fit2);

R = Fit(:,5); % pulling just observable signal

M1 = Fit(:,1);
M2 = Fit(:,2);
M3 = Fit(:,3);
M4 = Fit(:,4);
MSPR = Fit(:,5);

function dy = DiffEq1(t,y)
%answer matrix
dydt=zeros(4,1);
%Unknown Constants
%k(1)    forward rate constant of step1 (1/Ms) State 1, Reversible
%k(2)    reverse rate constant of step2 (1/s)
%k(3)    forward rate constant of step1 (1/Ms) State 2, Irreversible
%k(4)    reverse rate constant of step2 (1/s)
%k(5)    lipid extraction term, coming from Reversible binding
%k(6)    lipid extraction term, coming from Irreversible binding
%k(7)    maximum amount of peptide that can be bound (M)
%Model equations
dydt(1) = k(1)*Cp*(k(7)-y(1)-y(2)-y(3)-y(4)) - k(2)*y(1) + k(4)*y(2) -
k(3)*y(1) - k(5)*y(1); % dR1/dt
```

```
dydt(2) = k(3)*y(1) - k(4)*y(2) - k(6)*y(2); % dR2/dt
dydt(3) = k(5)*y(1); % dR3/dt
dydt(4) = k(6)*y(2);
dydt(5) = dydt(1) + dydt(2); %total observed R, dR/dt
dy=dydt;
end

function dy = DiffEq2(t,y)
%answer matrix
dydt=zeros(4,1);
dydt(1) = k(1)*0*(k(7)-y(1)-y(2)-y(3)-y(4)) - k(2)*y(1) + k(4)*y(2) -
k(3)*y(1) - k(5)*y(1); % dR1/dt
dydt(2) = k(3)*y(1) - k(4)*y(2) - k(6)*y(2); % dR2/dt
dydt(3) = k(5)*y(1); % dR3/dt
dydt(4) = k(6)*y(2);
dydt(5) = dydt(1) + dydt(2); %total observed R, dR/dt
dy=dydt;
end
end
```

Supplementary References.

1. Brown, J. S. *et al.* Synthesis and Solution-Phase Characterization of Sulfonated Oligothioetheramides. *Macromolecules* **50,** 8731–8738 (2017).
2. Porel, M. & Alabi, C. A. Sequence-defined polymers via orthogonal allyl acrylamide building blocks. *J. Am. Chem. Soc.* **136,** 13162–13165 (2014).
3. Wang, C. Z. *et al.* Synthesis and characterization of cationic polymeric nanoparticles as simvastatin carriers for enhancing the osteogenesis of bone marrow mesenchymal stem cells. *J. Colloid Interface Sci.* **432,** 190–199 (2014).
4. Arbuse, A. *et al.* DNA-cleavage induced by new macrocyclic schiff base dinuclear Cu(I) complexes containing pyridyl pendant arms. *Inorg. Chem.* **48,** 11098–11107 (2009).
5. Porel, M., Thornlow, D. N., Artim, C. M. & Alabi, C. A. Sequence-Defined Backbone Modifications Regulate Antibacterial Activity of OligoTEAs. *ACS Chem. Biol.* **12,** 715–723 (2017).
6. Osby, J. O., Martin, M. G. & Ganem, B. An exceptionally mild deprotection of phthalimides. *Tetrahedron Lett.* **25,** 2093–2096 (1984).
7. Chen, H. C. & Chen, S. H. Diffusion of crown ethers in alcohols. *J. Phys. Chem.* **88,** 5118–5121 (1984).
8. Ortega, A. & García de la Torre, J. Hydrodynamic properties of rodlike and disklike particles in dilute solution. *J. Chem. Phys.* **119,** 9914 (2003).
9. Perrin, F. Mouvement brownien d'un ellipsoide - I. Dispersion diélectrique pour des molécules ellipsoidales. *J. Phys. le Radium* **5,** 497–511 (1934).
10. Perrin, F. Mouvement Brownien d'un ellipsoide (II). Rotation libre et dépolarisation des fluorescences. Translation et diffusion de molécules ellipsoidales. *J. Phys. le Radium* **7,** 1–11 (1936).
11. Koenig, S. H. Brownian Motion of an Ellipsoid: A correction to Perrin's Results. *Biopolymers* **14,** 2421–2423 (1975).
12. Macchioni, A., Ciancaleoni, G., Zuccaccia, C. & Zuccaccia, D. Determining accurate molecular sizes in solution through NMR diffusion spectroscopy. *Chem. Soc. Rev.* **37,** 479–489 (2008).
13. Svergun, D. I. Determination of the regularization parameter in indirect-transform methods using perceptual criteria. *J. Appl. Crystallogr.* **25,** 495–503 (1992).

14. Hopkins, J. B., Gillilan, R. E. & Skou, S. BioXTAS RAW: Improvements to a free open-source program for small-angle X-ray scattering data reduction and analysis. *J. Appl. Crystallogr.* **50,** 1545–1553 (2017).
15. Salzberg, L. I. & Helmann, J. D. Phenotypic and transcriptomic characterization of Bacillus subtilis mutants with grossly altered membrane composition. *J. Bacteriol.* **190,** 7797–7807 (2008).
16. Beining, P. R., Huff, E., Prescott, B. & Theodore, T. S. Characterization of the lipids of mesosomal vesicles and plasma membranes from Staphylococcus aureus. *J. Bacteriol.* **121,** 137–143 (1975).
17. Cheng, J. T. J., Hale, J. D., Elliott, M., Hancock, R. E. W. & Straus, S. K. The importance of bacterial membrane composition in the structure and function of aurein 2.2 and selected variants. *Biochim. Biophys. Acta - Biomembr.* **1808,** 622–633 (2011).
18. Mishra, N. N. & Bayer, A. S. Correlation of cell membrane lipid profiles with daptomycin resistance in methicillin-resistant staphylococcus aureus. *Antimicrob. Agents Chemother.* **57,** 1082–1085 (2013).
19. Haest, C. W. M., De Gier, J., Op Den Kamp,]J.A.F., Bartels, P. & Van Deenen, L. L. M. Changes in permeability of Staphylococcus aureus and derived liposomes with varying lipid composition. *Biochim. Biophys. Acta - Biomembr.* **255,** 720–733 (1972).
20. Yasuhiro, K., Tieko, Y. & Hideo, H. Alteration of the phospholipid composition of Staphylo-coccus aureus cultured in medium containing NaCl. *Biochim. Biophys. Acta - Lipids Lipid Metab.* **280,** 444–450 (1972).
21. García-Fernández, E. *et al.* Membrane Microdomain Disassembly Inhibits MRSA Antibiotic Resistance. *Cell* **171,** 1354–1367.e20 (2017).
22. Tsai, M. *et al.* Staphylococcus aureus requires cardiolipin for survival under conditions of high salinity. *BMC Microbiol.* **11,** (2011).
23. Mishra, N. N. *et al.* Carotenoid-related alteration of cell membrane fluidity impacts Staphylococcus aureus susceptibility to host defense peptides. *Antimicrob. Agents Chemother.* **55,** 526–531 (2011).
24. Hayami, M., Okabe, A., Kariyama, R. & Kanemasa, Y. Lipid Composition of Staphylococcus and Its Derived L-forms. *Microbiol. Immunol.* **23,** 435–442 (1979).
25. White, D. C. & Frerman, F. E. Extraction, Characterization, and Cellular Localization of the Lipids of Staphylococcus aureus. *J. Bacteriol.* **94,** 1854–1867 (1967).
26. White, D. C. & Frerman, F. E. Fatty acid composition of the complex lipids of Staphylococcus aureus during the formation of the membrane-bound electron transport system. *J. Bacteriol.* **95,** 2198–2209 (1968).
27. Short, S. A. & White, D. C. Metabolism of the glycosyl diglycerides and phosphatidylglucose of Staphylococcus aureus. *J. Bacteriol.* **104,** 126–32 (1970).
28. Hsia, C.-Y., Chen, L., Singh, R. R., DeLisa, M. P. & Daniel, S. A Molecularly Complete Planar Bacterial Outer Membrane Platform. *Sci. Rep.* **6,** 32715 (2016).
29. Zack, G. W., Rogers, W. E. & Latt, S. A. Automatic measurement of sister chromatid exchange frequency. *J. Histochem. Cytochem.* **25,** 741–53 (1977).
30. Hall, K., Lee, T.-H., Mechler, A. I., Swann, M. J. & Aguilar, M.-I. Real-time Measurement of Membrane Conformational States Induced by Antimicrobial Peptides: Balance Between Recovery and Lysis. *Sci. Rep.* **4,** 1–9 (2014).
