# Supplementary material for: Antibacterial isoamphipathic oligomers highlight the importance of multimeric lipid aggregation for antibacterial potency
Source: Commun Biol. 2018 Dec 7;1:220. doi: 10.1038/s42003-018-0230-4 (PMC6286309; doi:10.1038/s42003-018-0230-4)
Supplement: Supplementary file 2 — Description of Additional Supplementary Files [file 42003_2018_230_MOESM2_ESM.docx]

**Description of Additional Supplementary Files**

**File Name**: Supplementary Movie 1

**Description**: 5 μM Meta disrupting SLB membrane to generate aggregates. Time-lapse fluorescence microscopy of 5 μM Meta placed onto an R18-labeled S. aureus mimetic supported lipid bilayer (SLB). Time of oligomer exposure to membrane surface is indicated in black in the upper left corner in minutes:seconds. 100 μm scale bar provided.

**File Name**: Supplementary Movie 2

**Description**: 5 μM Para disrupting SLB membrane to generate more numerous smaller aggregates. Time-lapse fluorescence microscopy of 5 μM Para placed onto an R18-labeled S. aureus mimetic supported lipid bilayer (SLB). Time of oligomer exposure to membrane surface is indicated in black in the upper left corner in minutes:seconds. 100 μm scale bar provided.

**File Name**: Supplementary Movie 3

**Description**: 5 μM Para disrupting SLB membrane to generate aggregates with different lipid label. Time-lapse fluorescence microscopy of 5 μM Para placed onto a Texas RedTM-labeled S. aureus mimetic supported lipid bilayer (SLB). Time of oligomer exposure to membrane surface is indicated in black in the upper left corner in minutes:seconds. 100 μm scale bar provided.
